# Investigation into the role of the MITA-TRIM38 interaction in regulating pyroptosis and maintaining immune tolerance at the maternal-fetal interface

**DOI:** 10.1038/s41419-023-06314-w

**Published:** 2023-11-28

**Authors:** Jun Liu, Yan Deng, An Wang, Bowen Liu, Xi Zhou, Tailang Yin, Yan Wang, Tao Tang, Yang Qiu, Jiao Chen, Jing Yang

**Affiliations:** 1 Reproductive Medical Center, Renmin Hospital, Wuhan University, Wuhan, China; 2 Wuhan Children’s Hospital (Wuhan Maternal and Child Healthcare Hospital), Tongji Medical College, Huazhong University of Science & Technology, Wuhan, China; 3 Department of Obstetrics & Gynaecology, Faculty of Medicine, The Chinese University of Hong Kong, Shatin, Hong Kong; 4 State Key Laboratory of Virology, Wuhan Institute of Virology, Center for Biosafety Mega-Science, Chinese Academy of Sciences (CAS), Wuhan, China; 5 Institute of Hydrobiology, Chinese Academy of Sciences, Wuhan, China

**Keywords:** Cell division, Cell death, Cell death and immune response, Immune cell death

## Abstract

The maternal-fetal interface shares similarities with tumor tissues in terms of the immune microenvironment. Normal pregnancy is maintained due to the immunosuppressed state, but pyroptosis induced by MITA can trigger the body’s immune response and disrupt the immunosuppressed state of the maternal-fetal interface, leading to abortion. In this study, we explored the role of MITA and TRIM38 in regulating pyroptosis and maintaining the immune tolerance of the maternal-fetal interface during pregnancy. Our findings show that the interaction between MITA and TRIM38 plays a crucial role in maintaining the immunosuppressed state of the maternal-fetal interface. Specifically, we observed that TRIM38-mediated K48 ubiquitination of MITA was higher in M2 macrophages, leading to low expression levels of MITA and thus inhibiting pyroptosis. Conversely, in M1 macrophages, the ubiquitination of K48 was lower, resulting in higher expression levels of MITA and promoting pyroptosis. Our results also indicated that pyroptosis played an important role in hindering the transformation of M1 to M2 and maintaining the immunosuppressed state of the maternal-fetal interface. These discoveries help elucidate the mechanisms that support the preservation of the immune tolerance microenvironment at the maternal-fetal interface, playing a vital role in ensuring successful pregnancy.

## Introduction

Recurrent spontaneous abortion (RSA) is defined as two or more consecutive instances of spontaneous abortion in the same individual. Studies show that the incidence of RSA is 1–4% in women of childbearing age in Europe and the US [1], with 50% of cases occurring during the first trimester [2]. The causes of RSA are complex and diverse, including known factors such as chromosomal abnormalities, reproductive structural issues, endocrine disorders, infectious diseases, prethrombotic status, and autoimmune factors. Additionally, a substantial number of cases are referred to as unexplained RSA or URSA [3], where the cause remains unclear.

Pregnancy can be considered a semiallogenic transplantation process where the fetus survives, matures and develops without immune rejection, relying on the mother’s immune tolerance [4]. The maternal-fetal interface, composed of the placenta and decidua, is key in establishing immune tolerance and is the site of disturbance in many cases of poor pregnancy outcomes [5–7]. Macrophages, a crucial component in the course of pregnancy, play an important role in immune regulation, particularly subsets M1 and M2, whose dysregulation often results in adverse outcomes such as URSA and eclampsia [8, 9]. Macrophages are also known as major antigen-presenting cells, and their activation can stimulate the innate immune response [10], making them a popular subject of research.

MITA (also called stimulator of interferon genes, STING) is an adaptor protein that plays a crucial role in natural immunity [11]. This molecule recognizes both viral and bacterial infections, as well as its own DNA, triggering host defence and immune responses [11]. MITA is highly expressed in the heart, spleen, peripheral leukocytes, placenta, and lung and moderately expressed in the thymus, small intestine, liver, and kidney [12, 13]. However, this molecules is not expressed in the brain, skeletal muscle, or colon [12, 13]. The ubiquitin‒proteasome degradation pathway is important for intracellular selective protein degradation, and MITA can undergo ubiquitination through this pathway [14]. The TRIM family, mostly defined as E3 ubiquitin ligases, plays crucial roles in the ubiquitination of MITA [15, 16]. In tumor tissues, MITA is highly expressed in tumor-associated macrophages, and its activation can repolarize M2 TAMs into M1 TAMs [17]. In cancer cells, MITA expression is suppressed to help cancer cells evade the body’s immune surveillance [18]. The role of MITA and ubiquitination in the polarization of macrophages at the maternal-fetal interface, which forms an immune tolerance state similar to tumor tissue [19], is not well understood.

Pyroptosis is a type of programmed cell death that is mediated by gasdermin-D (GSDMD). During pyroptosis, cells experience increased swelling and develop vesicular protrusions [20]. The proteins of the cysteine aspartate specific proteinase (caspase) family, which are mainly activated by inflammasomes, cleave and activate GSDMD proteins [21]. Pro-caspase-1 (p20) is a key protein in pyroptosis and is a cleavage product of mature caspase-1 (p45) [22]. This protein cleaves GSDMD into GSDMD-N, which forms pores in the cell membrane and causes cell death from the inside [23]. However, the released GSDMD-N does not damage neighboring mammalian cells during pyroptosis due to its preference for lipid binding [23].

Activation of MITA can initiate pyroptosis and induce an immune response in macrophages [24, 25]. Previous research has shown that there is a higher rate of pyroptosis in the decidual tissue of patients with recurrent abortion than in that of healthy pregnant patients [26]. However, there is no information available on the relationship between the ubiquitination process and pyroptosis in macrophages at the maternal-fetal interface. In this study, we demonstrate that MITA can be degraded by type K48 ubiquitination, which is mediated by TRIM38 in M2 macrophages. The decreased expression of MITA leads to a decrease in pyroptosis, revealing a potential association between ubiquitination and pyroptosis. This phenomenon may be why the low expression of MITA results in maternal-fetal immune tolerance and helps to sustain pregnancy.

## Results

### Differential expression of pyroptosis-associated markers and macrophage subpopulations in decidual tissues between patients with URSA and control patients

**Fig. 1:**
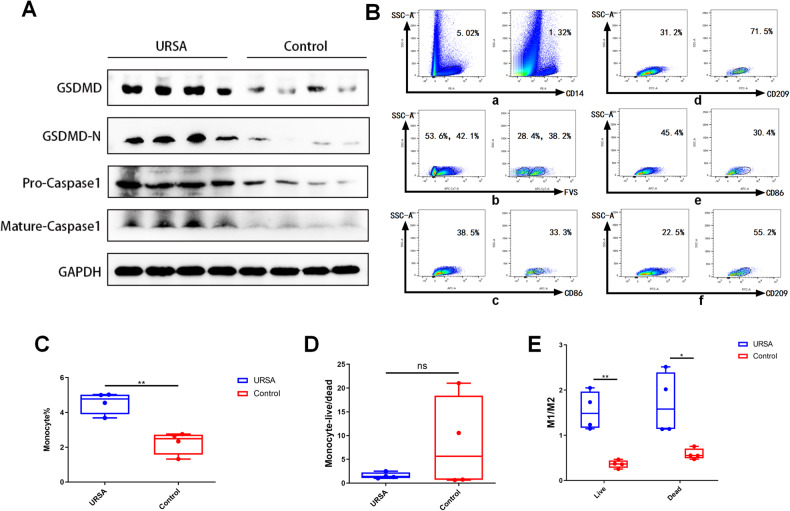
Differential expression of pyroptosis-associated markers and macrophage subpopulations in decidual tissues between patients with URSA and control patients. **A** Differences in the protein expression levels of GSDMD, GSDMD-N, pro-caspase-1 and mature caspase-1 in decidual tissues between patients in the URSA group and the control group. **B** FCM analysis shows the content of CD14-labeled macrophages, FVS-labeled live/dead cells, and the contents of M1 macrophages labeled by CD86 and M2 macrophages labeled in CD209 in viable and dead cells in the decidual tissues in one patient with URSA and one control patient. **a** shows that the percentages of all the macrophages with CD14 (+) in the URSA and control groups are 5.02% and 1.32%, respectively; **b** shows that the percentages of CD14 (+) in FVS (−) and FVS (+) in the URSA and control groups are 53.6%, 42.1%, 28.4% and 38.2%; **C** shows that the percentages of macrophages showing the FVS (−) survival status represented by CD86 (+) M1 macrophages in the URSA group and control group are 38.5% and 33.3%, respectively; **d** shows that the percentages of macrophages showing the FVS (−) survival status represented by CD209 ( + ) M2 macrophages in the URSA and control groups are 31.2% and 71.5%, respectively; **e** shows that the percentages of macrophages showing the FVS (+) dead status represented by CD86 (+) M1 macrophages in the URSA group and control group are 45.4% and 30.4%; **f** shows that the percentages of macrophages showing the FVS (+) dead status represented by CD209 (+) M2 macrophages in the URSA group and control group are 22.5% and 55.2%. **C** FCM analysis of differences in total macrophage content in decidual tissues between patients in the URSA group and the control group. **D** FCM analysis of the proportion of macrophage survival and death in the uterine decidual tissues of patients in the URSA group and the control group. **E** FCM analysis of the difference in the proportion of M1 and M2 macrophages in the surviving and dead macrophages of the URSA group and the control group. *P < 0.05, **P < 0.01, ns = nonsignificant.

Decidual tissues were obtained from four patients with unexplained recurrent spontaneous abortion (URSA) and four control patients who had a normal pregnancy but underwent induced abortion. Western blot analysis results demonstrated significantly higher expression of GSDMD, gasdermin-D-N (GSDMD-N), pro-caspase-1, and mature caspase-1 in the URSA group than in the control group (as depicted in Fig. 1A). Flow cytometry was then employed to assess various parameters in decidual tissues from both groups, including the overall proportion of macrophages (Fig. 1B-a), the proportion of surviving and dead macrophages (Fig. 1B-b), the proportion of surviving M1 and M2 macrophages (Fig. 1B-c, B-d), and the proportion of dead M1 and M2 macrophages (Fig. 1B-e, B-f). Further analysis revealed a significantly higher total number of macrophages in the URSA group than in the control group (Fig. 1C). However, the ratio of surviving to dead macrophages did not exhibit a significant difference between the two groups (Fig. 1D). Interestingly, the ratio of M1 to M2 macrophages was significantly higher in the URSA group than in the control group, as observed in both surviving and dead macrophages (Fig. 1E). These findings suggest that the differential expression of pyroptosis-associated markers between patients with URSA and control patients might be associated with alterations in the populations of M1 and M2 macrophages.

### Differential expression of TRIM38 and MITA in the decidual tissue of patients in the URSA group and control group

**Fig. 2:**
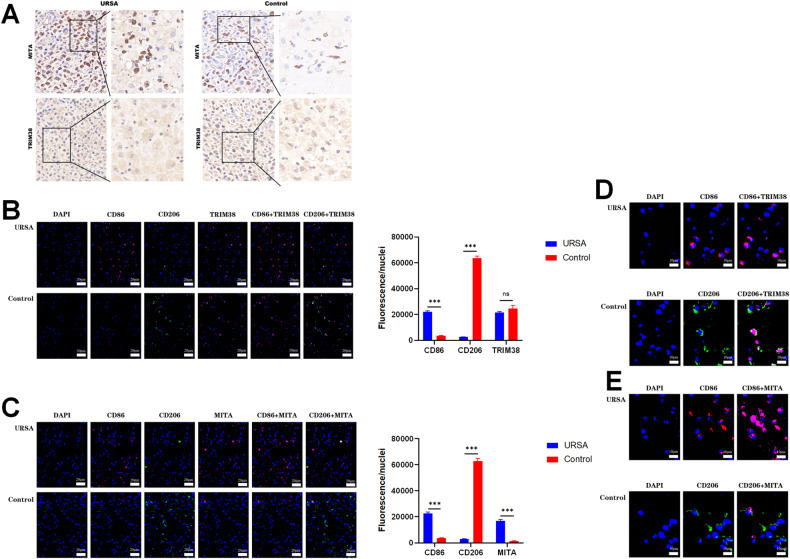
Differential expression of TRIM38 and MITA in decidual tissues between patients in the URSA group and the control group as determined by immunohistochemistry and immunofluorescence staining. **A** Immunohistochemistry shows the differences in MITA and TRIM38 expression in the uterine decidual tissue of patients with URSA and controls (brown part). **B** Triple immunofluorescence staining shows the distribution and expression differences of M1 macrophages (CD86 marker, red), M2 macrophages (CD206 marker, green) and TRIM38 (pink) in the uterine decidual tissue of patients with URSA and controls. **C** Immunofluorescence staining shows the differences in the distribution and expression differences of M1 macrophages (CD86 marker, red), M2 macrophages (CD206 marker, green) and MITA (pink) in the uterine decidual tissue of patients with URSA and controls. **D** Local magnification plot corresponding to **B**. **E** Local magnification plot corresponding to **C**.

The protein expression of TRIM38 and MITA in decidual tissues was evaluated using IHC. No difference in TRIM38 expression between the URSA and control groups was observed (as shown in Fig. 2A, B). However, MITA expression in decidual tissues was higher in the URSA group than in the control group (as shown in Fig. 2A, B). Triple IF analysis revealed that TRIM38 and MITA were specifically expressed in M1 macrophages (labeled with CD86, shown as red fluorescence) in the URSA group (Fig. 2C–F). In the control group, TRIM38 and MITA were mainly expressed in M2 macrophages (labeled with CD209, shown as green fluorescence), and MITA expression was significantly lower in M2 macrophages than in M1 macrophages in the URSA group (Fig. 2C–F).

**Fig. 3:**
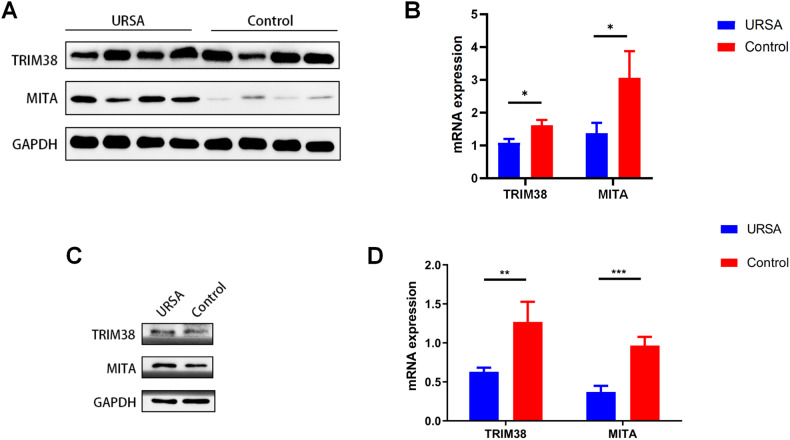
Differential expression of TRIM38 and MITA in the decidual tissue of patients in the URSA group and control group as determined by western blot and qPCR analyses. **A** Differences in the protein expression levels of TRIM38 and MITA in the decidual tissues of the URSA group and control group. **B** Differences in the mRNA expression levels of TRIM38 and MITA in the decidual tissues of the URSA group and control group. **C** Differences in the protein expression levels of TRIM38 and MITA in macrophages in decidual tissues from patients in the URSA group and control group. **D** Differences in the mRNA expression levels of TRIM38 and MITA in macrophages in decidual tissues from patients in the URSA group and control group. *P < 0.05, **P < 0.01, ***P < 0.001.

The expression of MITA protein in decidual tissues was higher in the URSA group than in the control group (Fig. 3A). In contrast, there was no significant difference in TRIM38 protein expression between the two groups (Fig. 3A). However, qRT-PCR results revealed that both TRIM38 and MITA mRNA expression in decidual tissues was decreased in the URSA group compared to the control group (Fig. 3B). These results were also supported by the macrophages obtained from decidual tissues (Fig. 3C, D).

### Differential ubiquitination of TRIM38 and MITA in decidual tissues and macrophages

**Fig. 4:**
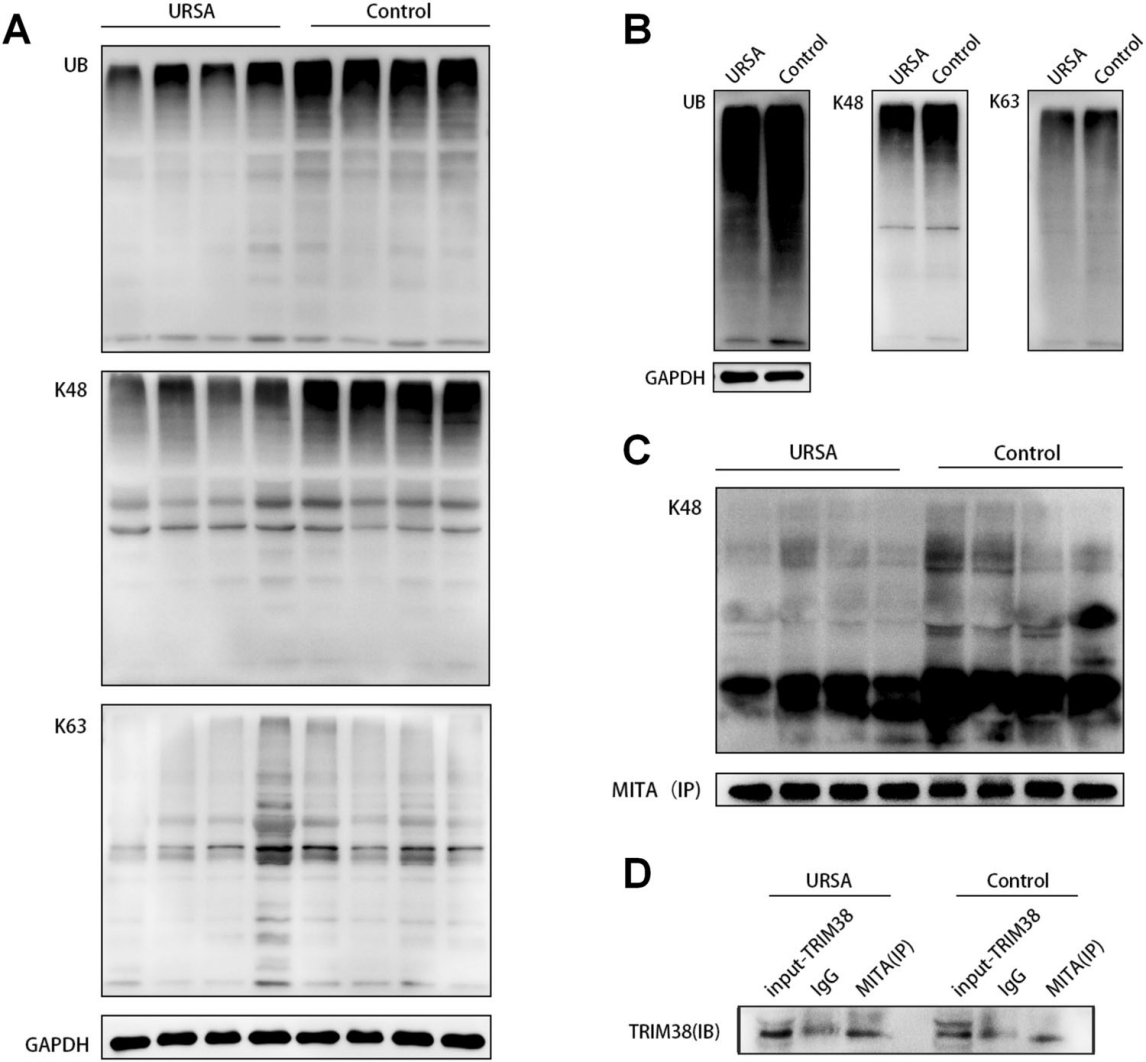
Different ubiquitination levels of UB, K48, and K63 occurred in the decidual tissues and macrophages of the URSA group and control group. **A** The higher expression of type UB and K48, but not K63, occurred in the decidual tissues of the control group compared to the URSA group. **B** The higher expression of type UB and K48, but not K63, occurred in macrophages of decidual tissues in the control group compared to the URSA group. **C** Higher expression of MITA by ubiquitination of type K48 in decidual tissues of the control group than in the URSA group. **D** Verification of the endogenous interaction relationship between TRIM38 and MITA in the decidual tissues of the URSA group and control group.

Additionally, we compared the levels of ubiquitination in decidual tissues and macrophages between the URSA and control groups. UB and K48 levels in decidual tissues and macrophages from the URSA group were significantly lower than those in the control group, while there was no significant difference in the level of K63 between the two groups (Fig. 4A, B). Furthermore, the level of K48 in MTIA was significantly lower in the URSA group than in the control group (Fig. 4C). Co-IP results indicated that there were endogenous interactions between TRIM38 and MITA in decidual tissues from both groups (Fig. 4D).

### Differential expression of pyroptosis-related proteins in the supernatants of M1 and M2 macrophages

**Fig. 5:**
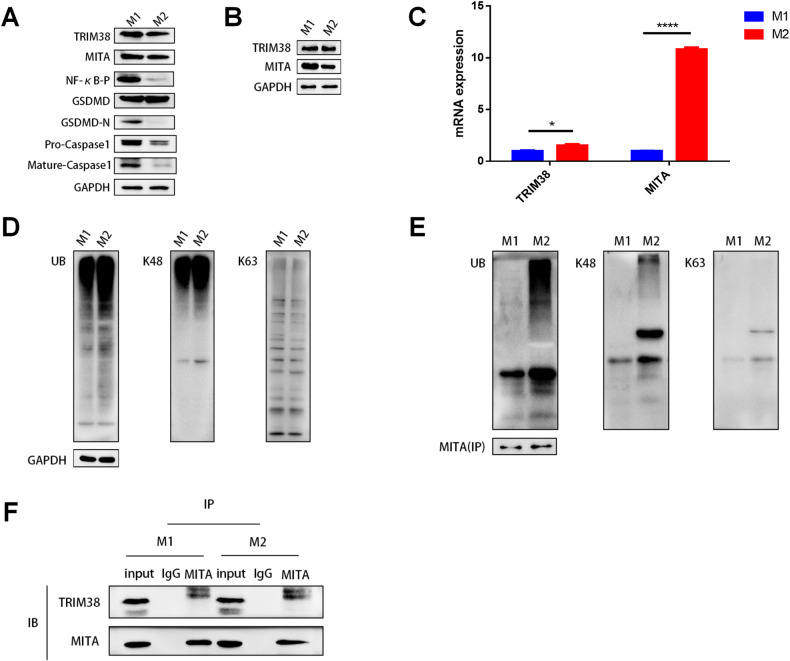
Differential expression of pyroptosis-related proteins in the supernatants and low M1/M2 cells in vitro. **A** Differences in the protein expression levels of TRIM38, MITA, GSDMD, GSDMD-N, pro-caspase-1 and mature caspase-1 in the supernatants of M1 and M2 macrophages. **B** Differences in the protein expression levels of TRIM38 and MITA in M1 and M2 macrophages. **C** Differences in the mRNA expression levels of TRIM38 and MITA in M1 and M2 macrophages. **D** The higher expression of type UB and K48, but not K63, occurred in M1 compared to M2 macrophages. **E** The higher expression of MITA by ubiquitination of types UB and K48, but not K63, occurred in M1 compared to M2 macrophages. **F** The endogenous interaction relationship between TRIM38 and MITA in M1 and M2 macrophages. *P < 0.05, **P < 0.01.

To validate our hypothesis, we created in vitro cell models using polarized M1 and M2 macrophages from THP-1 cells. Western blotting was used to analyze the protein expression of GSDMD, GSDMD-N, pro-caspase-1, and mature caspase-1 in the macrophage supernatants. GSDMD expression was lower, while the expression of GSDMD-N, pro-caspase-1, and mature caspase-1 was higher in the M1 supernatant than in the M2 supernatant (Fig. 5A). TRIM38 expression was also higher in the M1 supernatant, while MITA expression was not significantly different between the two (Fig. 2A). MITA expression was upregulated in M1 macrophages compared to M2 macrophages, but TRIM38 expression was not different (Fig. 5B). However, qRT‒PCR revealed lower TRIM38 and MITA mRNA expression in M1 macrophages than in M2 macrophages (Fig. 5C). The above results indicated that post-translational modifications might exist between TRIM38 and MITA.

### Differential expression and ubiquitination of TRIM38 and MITA in M1/M2 macrophages

The Co-IP results showed that there were endogenous interactions between TRIM38 and MITA in both M1 and M2 macrophages (Fig. 5F). Furthermore, we found higher expression of UB and K48, but not K63, in M2 macrophages than in M1 macrophages (Fig. 5D); higher ubiquitination of UB and K48, but not K63, also occurred in MITA in M1 macrophages than in M2 macrophages (Fig. 5E). Thus, based on the above results, we hypothesize that the ubiquitination of MITA may be related to pyroptosis in macrophages.

### TRIM38 containing the complete RING finger, B-box, and SPRY domains can interact not only with MITA but also with K48-type ubiquitinated particles

**Fig. 6:**
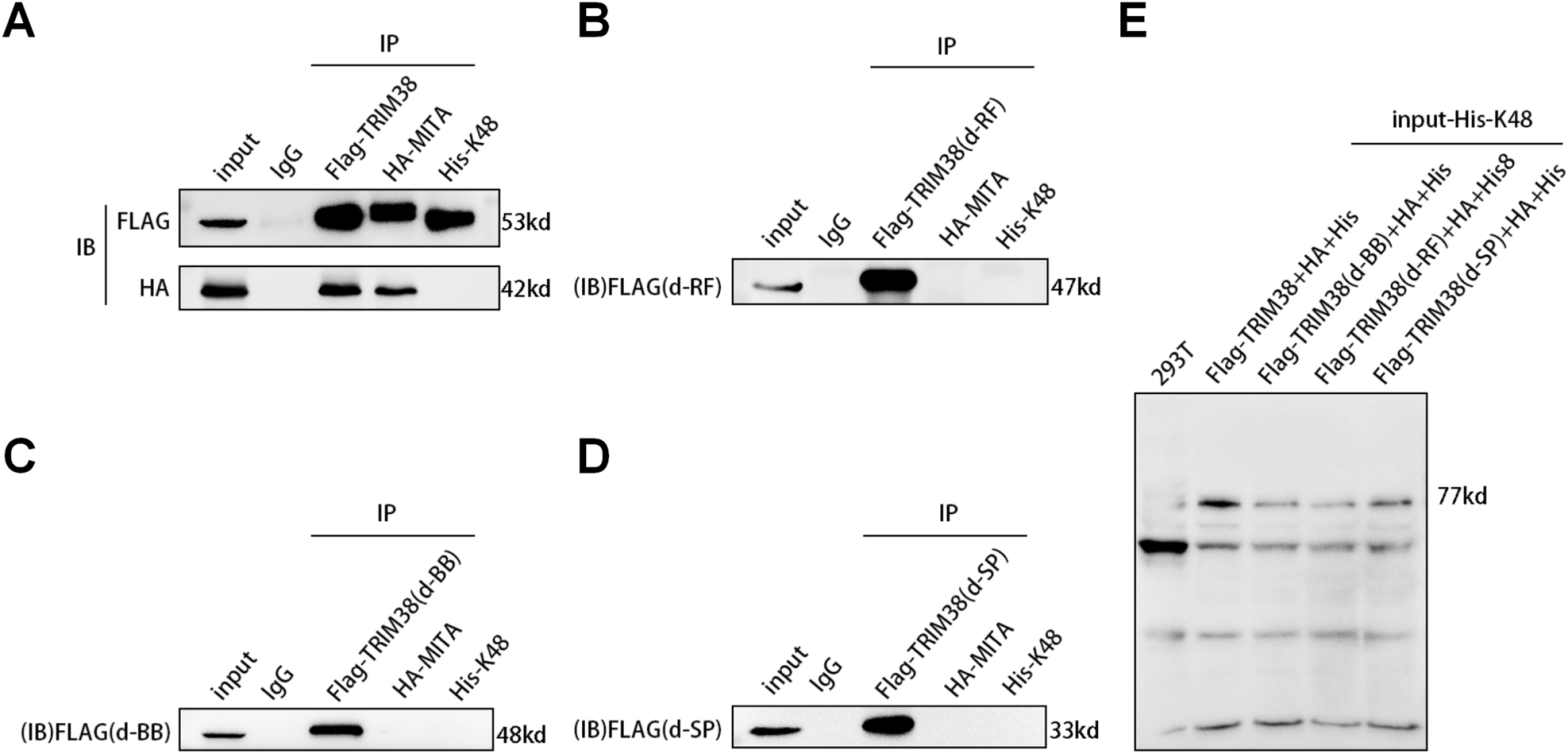
Exogenous interaction relationship among TRIM38 (Flag tag), MITA (HA tag) and K48 plasmid (His tag) in 293 T cells before and after knockdown of three domains. **A** Exogenous interaction relationship among TRIM38 (Flag tag), MITA (HA tag) and K48 plasmid (His tag). **B** Exogenous interaction relationship among knockdown of the ring finger structure of TRIM38 (Flag-TRIM38(d-RF)), MITA (HA-MITA) and K48 plasmid (His-K48). **C** Exogenous interaction relationship among knockdown of the B-box structure of TRIM38 (Flag-TRIM38(d-BB)), MITA (HA-MITA) and K48 plasmid (His-K48). **D** Exogenous interaction relationship among TRIM38 (Flag-TRIM38 (d-SP)), MITA (HA-MITA) and K48 plasmid (His-K48) with knockdown of the SPRY structure. **E** Expression validation of the type K48 ubiquitinated plasmid (His-K48) in 293 T cells (input).

As a member of the TRIM protein family, TRIM38 contains three functional domains: ring finger, B-box, and SPRY. To investigate the interplay among these three functional domains, MITA, and K48 ubiquitination, we cotransfected Flag-TRIM38, Flag-TRIM38 (d-RF), Flag-TRIM38 (d-BB), and Flag-TRIM38 (d-SP) with HA-MITA and His-K48 in 293 T cells. We observed that knocking down any of the three domains resulted in no interaction with HA-MITA (Fig. 6A–D). However, only TRIM38 with the complete ring finger, B-box, and SPRY domains was able to interact with His-K48 (Fig. 6A). If any of the three domains were knocked out, Flag-TRIM38 was unable to interact with HA-MITA and His-K48 (Fig. 6B–D). Furthermore, there was no direct interaction between HA-MITA and His-K48 (Fig. 6A–D).

### Inhibition of pyroptosis reduced MITA expression through enhanced K48 ubiquitination and promoted the conversion of M1 to M2 macrophages

**Fig. 7:**
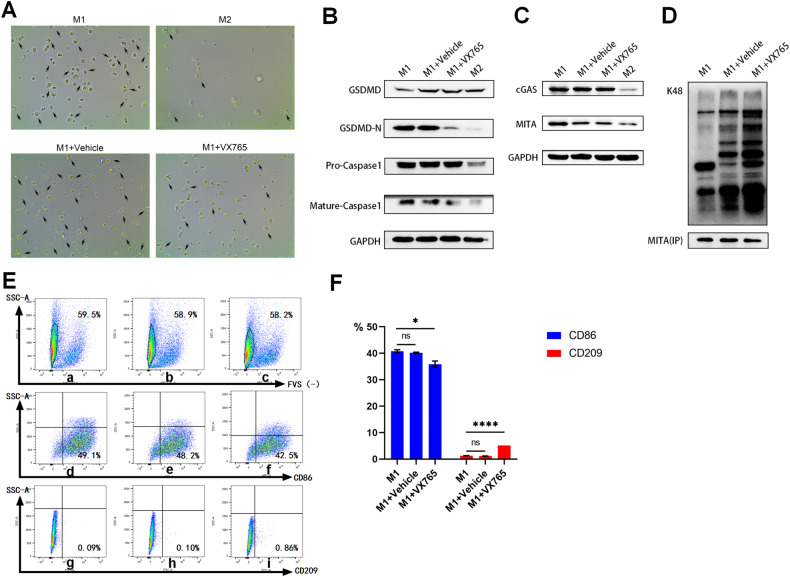
The relationship between pyroptosis and K48. **A** Morphology and number of pyroptotic cells in the supernatants of wild-type M1, M1 with DMSO (M1-vehicle), M1 with VX765 (M1-VX765) and wild-type M2 macrophages under a ×40 microscope. **B** Differences in the protein expression levels of GSDMD-N, caspase-1 and cleaved caspase-1 in the supernatants of M1, M1 with DMSO (M1-vehicle), M1 with VX765 (M1-VX765) and M2 macrophages. **C** Differences in the protein expression levels of MITA and cGAS in M1 macrophages, M1 macrophages treated with DMSO (M1-vehicle), M1 macrophages treated with VX765 (M1-VX765) and M2 macrophages. **D** Differential expression of MITA by ubiquitination of type K48 in M1, M1 with DMSO (M1-vehicle) and M1 with VX765 (M1-VX765) macrophages. **E** FCM analysis shows wild-type M1, M1 with DMSO (M1-vehicle), M1 with VX765 (M1-VX765) of FVS-labeled live cells, and the content of macrophages labeled by CD86 or CD209 in viable cells in the above three kinds of cells. **a**–**c** shows that the percentages of FVS (−) in M1, M1-vehicle and M1-VX765 are 59.5%, 58.9% and 58.2%, respectively. **d**–**f** shows that the percentages of macrophages showing the FVS (−) survival status represented by CD86 (+) macrophages in M1, M1-vehicle and M1-VX765 are 49.1%, 48.2% and 42.5%, respectively. **g**–**i** shows that the percentages of macrophages showing the FVS (−) survival status represented by CD209 (+) macrophages in M1, M1-vehicle and M1-VX765 are 0.09%, 0.10% and 0.86%, respectively. **F** The phenotypes of CD86 and CD209 in wild-type M1, M1-vehicle, and M1-VX765 cells were analyzed by FCM. *P < 0.05, ****P < 0.0001, ns = nonsignificant.

To investigate the relationship between ubiquitination and pyroptosis, we added belnacasan (VX765), a caspase-1 inhibitor, during polarization. The number of pyroptotic cells in the M1 + VX765 group was lower than that in the M1 + DMSO group but still higher than that in the M2 group (Fig. 7A). This finding suggests that pyroptosis in M1 macrophages is dependent on caspase-1. The expression of GSDMD-N and mature caspase-1 in the M1 + VX765 group was lower than that in the M1 group, while the expression of pro-caspase-1 was not significantly different (Fig. 7B). This result indicates that VX765 reduces the conversion of pro-caspase-1 to mature caspase-1.

We analyzed the protein expression levels of cGAS and MITA in macrophages and found that cGAS and MITA were significantly lower in M2 macrophages than in M1 macrophages (Fig. 7C). The expression of cGAS in the M1 + VX765 group was not significantly different from that in the M1 group, but the expression of MITA in the M1 + VX765 group was significantly lower (Fig. 7C). This finding suggests that VX765 can directly inhibit MITA expression without affecting cGAS, the upstream target of MITA. Furthermore, the level of K48 in MITA was significantly higher in the M1 + VX765 group than in the M1 group (Fig. 7D). Flow cytometry analysis showed that the number of CD86^+^ macrophages in the M1 + VX765 group decreased, while the number of CD209^+^ macrophages increased compared to that in the M1 group (Fig. 7E, F). These findings indicate that inhibiting pyroptosis reduces MITA expression through enhanced K48 ubiquitination and promotes the conversion of M1 to M2 macrophages.

### Effects of TRIM38 and MITA knockdown on the pyroptosis and polarization efficiency of macrophages

**Fig. 8:**
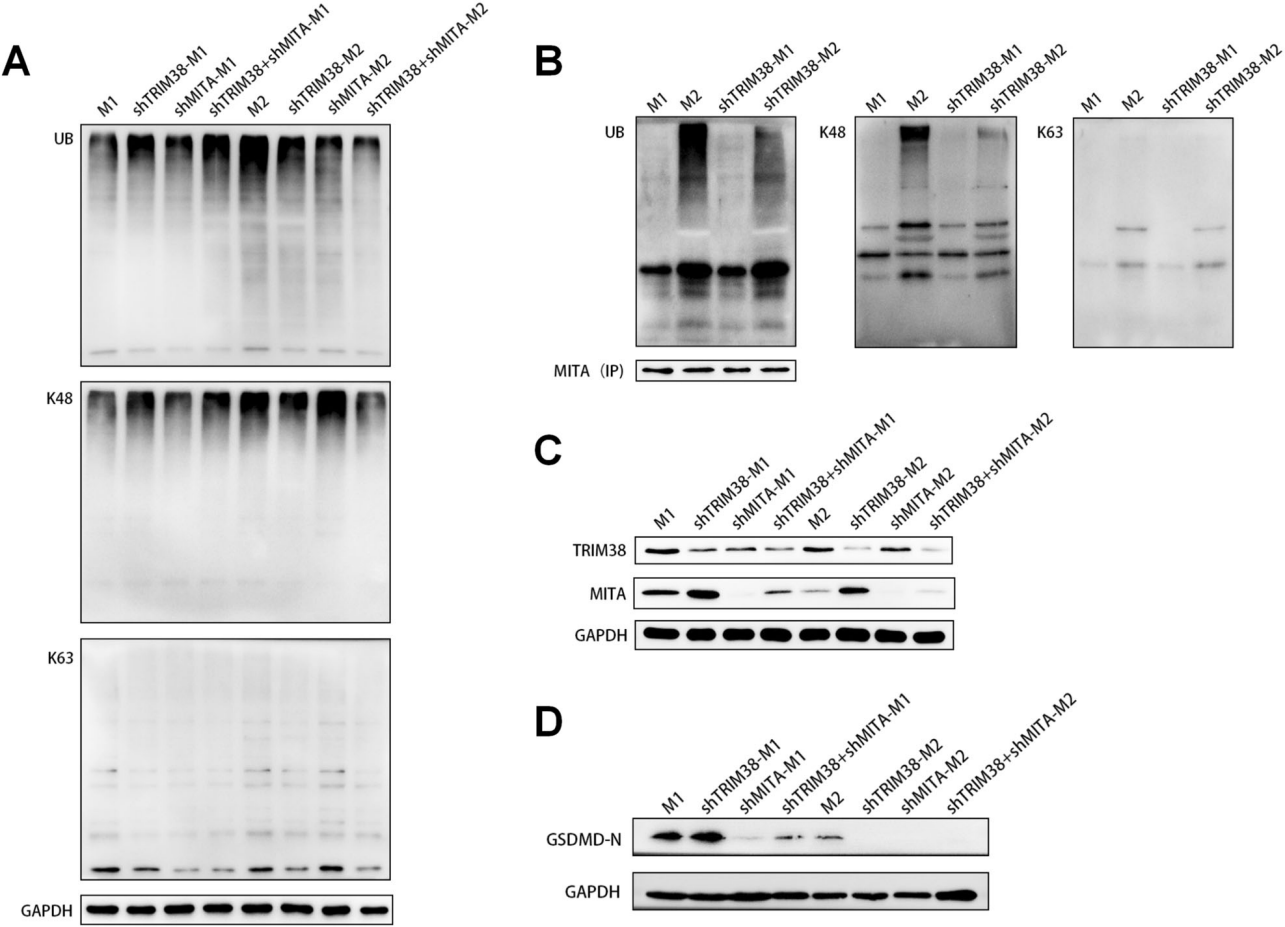
Lentiviral knockdown-related genes to verify the association among TRIM38, MITA, ubiquitination and pyroptosis. **A** Different ubiquitination levels of UB, K48, and K63 occurred in M1, shTRIM38-M1, shMITA-M1, shTRIM38+shMITA-M1 and M2, shTRIM38-M2, shMITA-M2, and shTRIM38+shMITA-M2 cells. **B** Differential expression of MITA by ubiquitination of types UB, K48, and K63 in M1, M2, shTRIM38-M1 and shTRIM38-M2. **C** Differences in the protein expression levels of TRIM38 and MITA in M1, shTRIM38-M1, shMITA-M1, shTRIM38+shMITA-M1 and M2, shTRIM38-M2, shMITA-M2, and shTRIM38+shMITA-M2 cells. **D** Differences in the protein expression levels of GSDMD-N in the supernatants of M1, shTRIM38-M1, shMITA-M1, shTRIM38+shMITA-M1 and M2, shTRIM38-M2, shMITA-M2, and shTRIM38+shMITA-M2 cells.

To investigate the impact of the K48 relationship among TRIM38, MITA, and pyroptosis, we created TRIM38 and MITA knockdown THP-1 cells (Supplemental Fig. S1). When TRIM38 was knocked down, the level of K48 was significantly higher in shTRIM38-M1 cells than in M1 cells but significantly lower in shTRIM38-M2 cells than in M2 cells (Fig. 8A). Conversely, the levels of K48 in shMITA-M1 and shMITA-M2 cells showed no significant difference compared to those of M1 and M2 cells, respectively (Fig. 8A). Additionally, when TRIM38 and MITA were both knocked down, the level of K48 in shTRIM38+shMITA-M1 cells was higher than that in M1 cells but significantly lower in shTRIM38+shMITA-M2 cells than in M2 cells (Fig. 8A). This finding suggests that TRIM38 suppresses K48 expression in M1 but enhances it in M2. Our findings also showed that the levels of K48 of MITA in shTRIM38-M1 cells were not significantly different from those in M1 cells (Fig. 8B), which could explain the increased expression of MITA in shTRIM38-M1 cells (Fig. 8C). Conversely, the levels of K48 of MITA in shTRIM38-M2 cells were significantly reduced compared to those in M2 cells (Fig. 8B), resulting in the increased expression of MITA in shTRIM38-M2 cells (Fig. 8C). These results indicated that TRIM38 directly regulated K48 of MITA in M2 but not in M1 macrophages.

Additionally, the levels of GSDMD-N in the shTRIM38-M1 supernatant were significantly higher than those in the M1 supernatant (Fig. 8D). This effect is closely correlated with the increased expression of MITA in shTRIM38-M1 cells. However, the levels of GSDMD-N in the shMITA-M1 supernatant were significantly lower than those in the M1 supernatant (Fig. 8D). In contrast, the levels of GSDMD-N in the shTRIM38 + shMITA-M1 supernatant were also significantly lower than those in the M1 supernatant but higher than those in the shMITA-M1 supernatant (Fig. 8D). Since GSDMD-N was rarely expressed in the M2 supernatant, almost no GSDMD-N could be detected in the supernatants of shTRIM38-M2, shMITA-M2, and shTRIM38 + shMITA-M2 cells (Fig. 8D). These results indicated that MITA could activate pyroptosis, while inhibition of MITA expression could reduce pyroptosis in M1 macrophages.

**Fig. 9:**
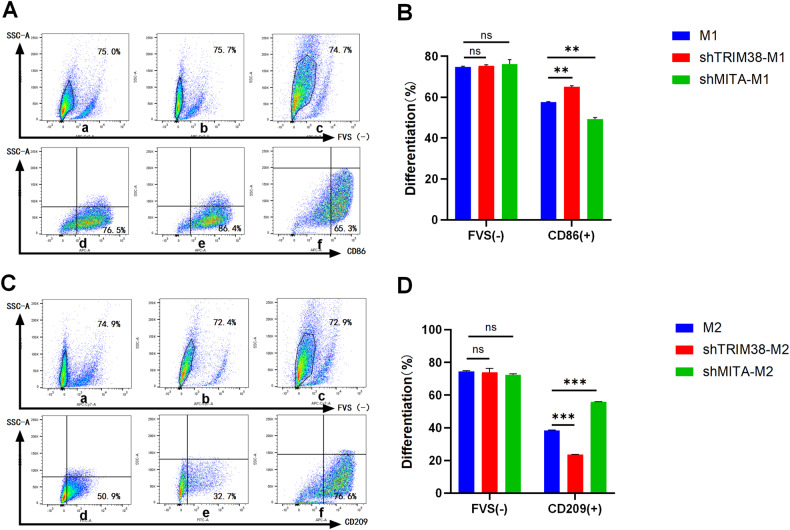
Effect of lentiviral knockdown of TRIM38 or MITA on the polarization efficiency of macrophages. **A** FCM analysis shows the wild-type M1, shTRIM38-M1, shMITA-M1 of FVS-labeled live cells, and the content of macrophages labeled by CD86 in viable cells in the above three kinds of cells, respectively. **a**–**c** shows the percentage of FVS (−) in M1, shTRIM38-M1, and shMITA-M1 are 75.0%, 75.7% and 74.7%, respectively. **d**–**f** shows that the percentages of macrophages showing the FVS (−) survival status represented by CD86 (+) macrophages in M1, shTRIM38-M1, and shMITA-M1 are 76.5%, 86.4% and 65.3%, respectively. **B** The phenotypes of FVS(−) and surviving macrophages with CD86(+) in wild-type M1, shTRIM38-M1, and shMITA-M1 cells were analyzed by FCM. **P < 0.01, ns = nonsignificant. **C** FCM analysis shows the wild-type M2, shTRIM38-M2, and shMITA-M2 FVS-labeled live cells and the content of macrophages labeled by CD209 in viable cells in the above three kinds of cells; **a**–**c** shows that the percentages of FVS (−) in M2, shTRIM38-M2, and shMITA-M2 are 74.9%, 72.4% and 72.9%, respectively; **d**–**f** shows the percentage of macrophages showing the FVS (−) survival status represented by CD209 (+) macrophages in M2, shTRIM38-M2, and shMITA-M2 are 50.9%, 32.7% and 76.6%, respectively. **D** The phenotypes of FVS(−) and surviving macrophages with CD209(+) in wild-type M2, shTRIM38-M2, and shMITA-M2 cells were analyzed by FCM. ***P < 0.001, ns nonsignificant.

When TRIM38 or MITA was individually knocked down, the polarization efficiency of shTRIM38-M1 was significantly higher than that of M1, whereas the polarization efficiency of shMITA-M1 was significantly lower than that of M1 (Fig. 9A, B). Conversely, the polarization efficiency of shTRIM38-M2 was significantly lower than that of M2, while the polarization efficiency of shMITA-M2 was significantly higher than that of M2 (Fig. 9C, D).

## Discussion

Toll like receptors (TLRs) are a type of pattern recognition receptors (PRRs) [27–29], mainly expressed on the surface of macrophages and dendritic cells. TLRs could bind five important aptamers MITA, TRIF, MyD88,VISA and ASC respectively, which relies on a series of E3 ubiquitin connection Enzyme-mediated ubiquitination [30]. It is currently believed that the ubiquitination of the adaptor protein MITA is a key to regulate TLRs signal activation in surface of macrophages [31]. TRIM38 belongs to the ubiquitination ligase family and is one of the most important ubiquitination regulator enzymes in TLRs signaling. Recent studies have shown that TRIM38 is mainly produced by TLR signal stimulation and is dependent on the activation of the NF-kB signaling pathway, serving as a negative feedback regulator of TLR signaling [31, 32]. Further research has confirmed that overexpression of TRIM38 can induce ubiquitination of the adaptor protein MITA, while the mutant TRIM38 (mut) with no ubiquitination connectivity activity does not mediate ubiquitination of MITA, which suggests that TRIM38 regulates immune signal transduction by regulating MITA ubiquitination. In macrophages, TRIM38 mediates the ubiquitination of MITA, accelerating the degradation of TRAF6 after TLR4 activation, thereby promoting the activation of IRF3/4/5 and others [33]. However, how TRIM38 regulates macrophage function by ubiquitination of MITA, how MITA ubiquitination level effects on the activation status of macrophages and the imbalance of immune at the maternal fetal interface, there still are no relevant reports. In this study, we found that the expression of MITA and pyroptosis-related proteins was significantly higher in the URSA group than in the control group, both in decidual tissues and macrophages. The control group also had significantly higher K48-type ubiquitination mediated by TRIM38, which may contribute to the lower expression of MITA. Using in vitro cell models, we investigated the relationship between K48 of MITA and pyroptosis-related proteins and revealed that K48 may play a role in maintaining normal pregnancy by reducing MITA expression.

The current focus of URSA research is on immune cells at the maternal-fetal interface, including natural killer (NK) cells, macrophages, and T lymphocytes. Macrophages are the predominant endometrial leukocytes and can be polarized into two types: classically activated (M1) and selectively activated (M2) [6, 34, 35]. Single-cell sequencing studies have shown that macrophages are the immune cell population that changes the most with pregnancy status [8]. An imbalance in the M1/M2 ratio in decidual tissue has been identified as a factor in URSA. In patients with URSA, M1 plays a dominant role in inflammation, while M2 helps regulate the immune response in normal pregnancy [36, 37]. The immune environment at the maternal-fetal interface is dynamic and regulatory, and decidual macrophages help maintain this environment by removing dead trophoblasts [38, 39]. However, the specific mechanisms and factors affecting macrophage polarization remain unclear.

MITA is a crucial adaptor protein in the innate immune system and plays a role in various diseases [40]. Low expression of MITA in tumor tissues has been linked to an immunosuppressed state that helps avoid the body’s immune response [18]. The maternal decidua, with similarities to the formation of cancer cell metastases, has also been studied [19]. TRIM38, a small molecule protein in the TRIM protein family, has three functional domains (ring finger, B-box, and SPRY) and is classified as an E3 ubiquitin ligase due to its ring finger structure. This molecule mediates various types of ubiquitination [28]. In this study, we found that the expression of MITA was significantly lower in normal pregnancy and M2 macrophages than in URSA and M1 macrophages. We also discovered the direct interaction between TRIM38 and MITA and the possibility of post-translational modification, such as K48-type ubiquitination, based on the different expression trends at the mRNA and protein levels. Ubiquitination, as an important post-translational modification [41], can manifest either as K48, which degrades the target protein, or K63, which causes structural changes in the target protein [42]. We found that K48 was higher in M2 than in M1 and that MITA ubiquitination by K48 was also higher in M2 than in M1. Our in vitro experiments showed that knocking down TRIM38 suppressed the intracellular ubiquitination level of M1 macrophages and promoted it in M2 macrophages. Furthermore, our findings indicate that downregulation of TRIM38 expression can enhance the polarization efficiency of M1 macrophages but suppress the polarization efficiency of M2 macrophages. Conversely, a reduction in MITA expression inhibits M1 polarization while promoting the polarization efficiency of M2 macrophages. This finding might explain why MITA has a low expression in M2 and suggests that MITA and TRIM38 do not significantly interact through ubiquitination in M1. Moreover, given the different functional statuses exhibited by TRIM38-MITA in M1 and M2, we speculated that TRIM38 may be structurally different between M1 and M2. However, by validation in 293 T cells, we found that when one of the above three domains was absent, TRIM38 could not mediate K48. The molecular weight of TRIM38 we detected either in vivo or in vitro was not consistent with the above defective TRIM38, from which we inferred that the different effects of TRIM38-MITA in M1 and M2 were not caused by the defective TRIM38.

In this study, we investigated whether pyroptosis mediated by MITA was involved in the ubiquitination process described above. This result was prompted by the different interaction functions displayed by MITA and TRIM38 in M1 and M2, as well as the fact that several members of the TRIM protein family can positively or negatively regulate cell pyroptosis [43]. Our findings showed that M1 macrophages underwent classical, caspase-1-dependent pyroptosis. Although previous studies have suggested that cell self-death can activate the cGAS-MITA pathway, leading to increased MITA production and a strong immune response [44], the activation of GSDMD-N could inhibit the cGAS-MITA pathway by promoting K+ efflux and reducing cGAS expression, ultimately leading to decreased MITA expression [45]. In this study, we found that the knockdown of MITA inhibited the production of GSDMD-N in M1, while the knockdown of TRIM38 increased the expression of GSDMD-N. This finding suggests that MITA promotes while TRIM38 suppresses pyroptosis in M1, confirming that TRIM38 and MITA play different roles in M1 and M2 and that this difference is closely related to their different cellular pyroptosis states. Interestingly, when we introduced VX765, an inhibitor of caspase-1, we found that both the levels of GSDMD-N in the supernatant and MITA expression in the cells were significantly decreased, regardless of cGAS expression. This result suggests that blocking pyroptosis could directly downregulate MITA expression and cause the transformation of M1 to M2, leading to an increase in the ubiquitinated degradation of MITA K48. This result also implies that MITA positively regulates pyroptosis in M1 macrophages, and the different pyroptosis states in M1 and M2 macrophages are likely the main reasons for the different interaction functions of MITA and TRIM38.

The validation of the tissue samples further confirmed the differential expression of MITA in decidual tissues in URSA and normal pregnancies, which was closely correlated with the proportion of M1 and M2 macrophages. While it has been reported that dead cells can release intracellular mitochondria and activate MITA-related pathways, leading to increased expression of MITA [46], our study found no clear differences in the proportion of surviving and dead macrophages between the two groups via FCM. IHC and triple IF also indicated that higher MITA expression in decidual tissues in the URSA group than the control group was strongly associated with viable M1 and M2, not dead macrophages. Additionally, we observed much higher K48-type ubiquitination in decidual tissue and macrophages from normal pregnancies than URSA in vivo, which was highly consistent with our cell models in vitro and with the M1/M2 ratio demonstrated by FCM, IHC, and triple IF.

## Conclusions

**Fig. 10:**
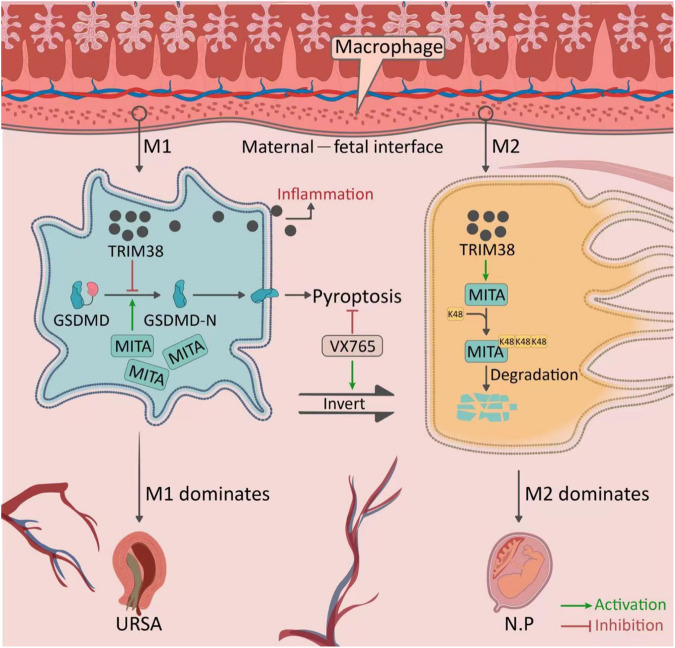
Pattern diagram. In macrophages at the maternal-fetal interface, when M1 macrophages were dominant, the high expression of MITA promoted the conversion of GSDMD to GSDMD-N, thus promoting pyroptosis, manifested as a proinflammatory effect, which led to URSA. When M2 macrophages were dominant, MITA mediated by TRIM38 led to low expression, which suppressed pyroptosis, manifested by an anti-inflammatory effect and subsequent maintenance of normal pregnancy (N.P.). However, when pyroptosis was inhibited by VX765, it could cause the conversion of M1 to M2 macrophages.

In this study, we found that low expression of MITA in M2 macrophages helps to avoid triggering an excessive immune response and maintain stability in the immune tolerant microenvironment at the maternal-fetal interface, enabling pregnancy to continue. In contrast, no effect of K48 on MITA was observed in M1 macrophages. Clear cell pyroptosis was observed in M1 macrophages, and when cell pyroptosis was inhibited, M1 macrophages not only exhibited K48 of MITA but also showed a clear trend towards transforming into M2 macrophages (Fig. 10). This finding suggests that cell pyroptosis may have an inhibitory effect on K48 of MITA and impact the transformation between M1 and M2 macrophages.

## Materials and methods

### Collection of clinical samples

Patients were aged 20–35 years, with menopause for 40–60 days, and were HCG (+); early uterine ultrasound found the original pericardial beats of the pregnant sac but the disappearance in the later stage, which indicated missed abortion, and the patients had a history of one or more unexplained abortions in the past. Four uterine decidual tissue samples without gene chromosomal abnormalities were taken as the URSA group, and four patients with uterine decidual tissue from early normal pregnancy (indicating the gestational sac and original cardiac beats) requiring artificial abortion to terminate pregnancy were used as the control group. The clinical characteristics of the included participants are shown in Table 1.

**Table 1:** Clinical characteristics of the included participants.

	URSA				Control			
	Patient1	Patient2	Patient3	Patient4	Patient1	Patient2	Patient3	Patient4
Age (years)	25	32	29	30	22	33	26	23
Race	Han	Han	Han	Han	Han	Han	Han	Han
Gestation	G2P0	G2P0	G2P0	G2P0	G2P0	G2P0	G2P0	G2P0
Menopause (days)	52	55	58	54	45	55	48	59
Past RSA	1	1	1	1	0	0	0	0
Disease history	No	No	No	No	No	No	No	No
Primary heartbeat	No	No	No	No	Yes	Yes	Yes	Yes
Villus CMV	(−)	(−)	(−)	(−)	(−)	(−)	(−)	(−)
COVID-19	(−)	(−)	(−)	(−)	(−)	(−)	(−)	(−)

Patients were aged 20–35 years, with menopause for 40–60 days, and were HCG (+); early uterine ultrasound found the original pericardial beats of the pregnant sac but the disappearance in the later stage, which indicated missed abortion, and the patients had a history of one or more unexplained abortions in the past. Four uterine decidual tissue samples without gene chromosomal abnormalities were taken as the URSA group, and four patients with uterine decidual tissue from early normal pregnancy (indicating the gestational sac and original cardiac beats) requiring artificial abortion to terminate pregnancy were used as the control group.

### Isolation of macrophages from tissue samples

Macrophages were isolated from decidual tissue using the conventional adherent method. Tissue samples (weighing approximately 5–8 g) from each patient were washed twice with sterile phosphate-buffered saline (PBS, Gibco) to remove visible blood clots. The tissue was sheared beforehand and added to a 10 cm sterile cell culture dish along with 2 ml of pancreatic enzyme analog (ATV, TrypLE™Express) at 37 °C for 15 mins in a thermostatic cell incubator. Then, 50 ml sterile centrifuge tubes were prepared, each corresponding to a patient’s tissue sample and marked separately. A 70 μm cell filter (Biosharp, Labgic, Beijing, China) was placed at the mouth of each tube. The ATV-digested decidual tissue was removed from the cell incubator, placed on the corresponding filter screen, and ground with 5 ml of sterile syringe and PBS. The cell suspension was collected in a centrifuge tube under the filter screen. After collection, a volume of 40 ml of PBS was added, thoroughly mixed, and then centrifuged at room temperature for 5 min at 1500 rpm. The resulting supernatant was discarded, and another 40 ml of PBS was added, mixed thoroughly, and centrifuged again at 1500 rpm for 5 min at room temperature. Once again, the supernatant was discarded, and 15 ml of PBS was added. Human red blood cell lysates, previously diluted to 1× with sterilized ddH2O, were added to the prepared cell suspension at a 1:1 ratio, thoroughly mixed at room temperature, and then placed in a black light protection box for 15 min. Next, the cell suspension was removed and centrifuged at 1500 rpm for 5 min at room temperature, and the supernatant was discarded. Subsequently, 40 ml of PBS was added, thoroughly mixed, and centrifuged at 1500 rpm for 5 min at room temperature. Once again, the supernatant was discarded. Based on the amount of cell precipitation in the lower layer, the cells were resuspended in 8–10 ml of complete medium prepared with Gibco RPMI 1640 medium + 10% Gibco fetal calf serum (FBS) + 1% double-antibody (penicillin + streptomycin, PS) + 1% 4-2-hydroxyethyl-1-ethylonic acid (HEPES). The cell count was measured, and 2 ml of the cell suspension was spread into a six-well plate for each patient sample. After a period of 6 h, the RPMI 1640 complete medium was replaced, and cell adhesion was observed using a microscope. The cells were replaced every 1–2 days thereafter and collected after 5–7 days. The entire process of sample collection was completed in the biosafety cabinet. After the upper cell medium was discarded, 2 ml of PBS was added to each well, and the attached macrophages were rinsed by gently shaking the plate. Next, 300 µl of ATV was added, and the surface was sprayed with 75% alcohol sterilized and digested in a 37 °C constant static cell incubator for 15 min. After the adherent cells became suspended under the 40x light microscope, digestion was terminated by adding sterile PBS (1 ml) to each well, and the cell suspension was transferred to a new sterile EP tube. Because there were fewer cells in each patient under the light microscope, cell samples from 4 patients in the URSA group and 4 patients in the control group were combined.

### Cell culture and polarization and VX765 treatment

The THP-1 cell line was a gift from Professor Xi Zhou (LRV-Group, Wuhan Institute of Virology, Chinese Academy of Sciences, Wuhan, China). THP-1 cells were cultured in RPMI 1640 medium (Gibco, Thermo Fisher, USA) with 10% fetal bovine serum (Gibco, Thermo Fisher, USA), 1% penicillin, 1% streptomycin and 1% 4-(2-hydroxyethyl)-1-piperazine ethylene sulfonic acid (Gibco, Thermo Fisher Scientific, USA) in an incubator containing 5% CO_2_ at 37 °C. Cell polarization was induced by incubating THP-1 cells (10^6^/ml) with phorbol 12-myristate 13-acetate (PMA) at a final concentration of 100 ng/ml (#P1585, Sigma, USA) for a 24-h period. This treatment caused the THP-1 cells to differentiate into M0 macrophages. For further polarization of the M0 macrophages, interferon-γ (IFN-γ) at a concentration of 20 ng/ml (#285-IF, R&D Systems, USA), lipopolysaccharide (LPS) at 10 pg/ml (#L2630, Sigma, USA), interleukin 4 (IL-4) at 20 ng/ml (#204-IL, R&D Systems, USA) and interleukin 13 (IL-13) at 20 ng/ml (#213-ILB, R&D Systems, USA) were added to the complete medium for 48 h, resulting in the polarization of M0 into M1 and M2 macrophages.

In addition, during the polarization of M1 macrophages, the caspase-1 inhibitor VX765 (#HY13205, MCE, working concentration: 25 µM) and equal amounts of dimethyl sulfoxide (DMSO, the solvent of VX765, 2.5 µl/10 ml) were added, together with IFN-γ and LPS.

### Knockdown of TRIM38 and MITA in THP-1 cells

Lentiviral particles were used to knockdown the TRIM38 and MITA genes in THP-1 cells. The particles targeting TRIM38 were obtained from Santa Cruz Biotechnology (#sc-95352-V, USA) and had a pool sequence of GATCCGTACAGATTCAGAGACAAATTCAAGAGATTTGTCTCTGAATCTGTACTTTTT + GATCCGTAGACTGAGGGACTATGATTCAAGAGATCATAGTCCCTCAGTCTACTTTT + GATCCCTGTCTCCTTGGAACTTCATTCAAGAGATGAAGTTCCAAGGAGACAGTTTTT, with a titer of 106 TU/ml. The particles targeting MITA were obtained from GenePharma (#D01001, China) and had a sequence of GCTGTCCATCTATTTCTACTA, with a titer of 108 TU/ml. The vector used for the MITA particles contained GFP green fluorescence. Blank lentiviral particles were obtained from GenePharma (#D03JZ, China). Transduction was carried out with a multiplicity of infection (MOI) of 1:100, as per the manufacturer’s instructions. Knockdown efficiency was confirmed through qRT‒PCR and western blot analysis. THP-1 cells were polarized into M1 and M2 macrophages after knockdown of TRIM38 or MITA and labeled shTRIM38-M1, shTRIM38-M2, shMITA-M1, and shMITA-M2. When both TRIM38 and MITA were knocked down, THP-1 cells were polarized into M1 and M2 macrophages and labeled shTRIM38+shMITA-M1 and shTRIM38+shMITA-M2, respectively.

### Transfection of 293 T cells

The 293 T cell line was provided as a gift from Professor Xi Zhou of LRV-Group, Wuhan Institute of Virology, Chinese Academy of Sciences, Wuhan, China, cultured in DMEM (Gibco, Thermo Fisher Scientific, USA) and maintained in an incubator at 37 °C with 5% CO2. The following plasmids were established by Miaolingbio, China: Flag-tagged TRIM38 plasmid (Flag-TRIM38), Flag-tagged TRIM38 plasmid without the ring finger domain (Flag-TRIM38 (d-RF)), Flag-tagged TRIM38 plasmid without the B-box domain (Flag-TRIM38 (d-BB)), Flag-tagged TRIM38 plasmid without the SPRY domain (Flag-TRIM38 (d-SP)), HA-tagged MITA plasmid (HA-MITA), and His-tagged K48 ubiquitination plasmid (His-K48). Flag-TRIM38, Flag-TRIM38 (d-RF), Flag-TRIM38 (d-BB), and Flag-TRIM38 (d-SP) were cotransfected with 8 μg of HA-MITA and His-K48 plasmids using Opti-MEM (Gibco, Thermo Fisher Scientific, USA) and PEI MAX (SenGene, Shanghai, China), following the manufacturer’s instructions. After 6–8 h of transfection, the medium was replaced with fresh DMEM, and the cells were harvested after incubation for 24 h.

### Flow cytometry and cell sorting

One milliliter of each tissue sample suspension was treated with red cell lysate and resuspended in 40 mL of PBS. Subsequently, the samples underwent centrifugation at a speed of 1500 rpm for 5 min. Following the removal of the supernatant, each tube was supplemented with 0.3–0.5% BSA (#V900933, Merck, USA) in PBS, which had been precooled to 4 °C, to resuspend the cells. The cell suspension was then subjected to centrifugation at 500 × *g* and 4 °C for 5 min, and this process was repeated once more after discarding the supernatant. The cells were resuspended in PBS to 200 µL, and the cell density was adjusted to 10^6^/mL by cell counting. The cell suspension was then passed through a 40 µm cell filter (Biosharp, Labgic, Beijing, China) into a new 1.5 mL EP tube. The filtered cell suspensions were divided into negative control (20 µL), CD14 (20 µL), FVS780 (20 µL), CD86 (20 µL), CD209 (20 µL), and sample tubes (100 µL). The negative control tube had PBS directly added to the resuspension to 300 µL, and the other tubes were filled with the recommended staining ratio (5 µL/100 µL cell suspension). FVS780 (20 µL) with 0.1 µL (1 µL/1000 µL cell suspension), 5 µL of the flow cytometry antibodies CD14 (#12-0149-42; PE channel, BD Biosciences, USA)/CD86 (#305412; APC channel, Biolegend, USA)/CD209 (#330104; FITC channel, Biolegend, USA) and 0.1 µL of FVS780 (#565388; APC-CY7 channel, BD Biosciences, USA) were added to the sample tubes (100 µL). The cell suspension in the above EP tubes was fully mixed and then placed in a fully sealed black light avoidance box for staining at room temperature for 30 min. After staining, 0.3–0.5% BSA in PBS precooled at 4 °C was added to each tube to resuspend the cells. The cell suspension was centrifuged at 500 × *g* and 4 °C for 5 min, and the operation was repeated once after discarding the supernatant. The cell suspension of the above EP tubes was resuspended with PBS to 300 µL, placed on ice together with the negative control to protect from light, and then analyzed by flow cytometry with a FACSAriaTM III (BD Biosciences). Data analysis was performed using FlowJo V10.8.0 software.

THP-1 cells and THP-1 cells with TRIM38 and MITA knockdown were seeded in T75 flasks at a density of 2.5 × 10^6^ cells per flask and differentiated with PMA. After incubation with either IFN-γ and LPS or IL-4 and IL-13, the cells were washed with cold PBS. The macrophages derived from M1 polarization were stained with flow antibodies against FVS780 and CD86, while M2 macrophages were stained with FVS780 and CD209 following the same flow steps as for the tissue. Since the lentiviral particle vector for knocking down the MITA gene contained GFP green fluorescence, CD209 (#330108; APC channel, Biolegend) was used in M2 with MITA knockdown. In other M2 macrophages, CD209 (#330104; FITC channel, Biolegend, USA) was used. The cells were analyzed by flow cytometry with a FACSAriaTM III system (BD Biosciences), and data analysis was performed using FlowJo V10.8.0 software. M1 with FVS780(−) and CD86(+) and M2 with FVS780(−) and CD209(+) were collected in sterile 15 ml centrifuge tubes containing RPMI 1640 medium for subsequent experiments.

### Real-time quantitative PCR (qPCR)

The isolation of total RNA from tissues was carried out using TRIzol reagent (TaKaRa, Japan), while cells were isolated using a kit from Sangon Biotech (Shanghai, China). The RNA was then reverse-transcribed using a high-capacity cDNA reverse transcriptase kit (TaKaRa, Japan) according to the manufacturer’s instructions. qPCR assays were conducted using SYBR Green PCR Master Mix (TaKaRa, Japan) and the Fast qPCR System (QuantStudio5, Thermo Fisher, USA). Gene-specific primers were designed and obtained from Ruibo Biology Co., Ltd. (Shanghai, China). The primer sequences can be found in Table 2. For normalization of gene expression levels, glyceraldehyde-3-phosphate dehydrogenase (GAPDH) was chosen as the internal control. Three biological replicates were utilized for each sample. The 2^−ΔΔCt^ method was employed to calculate and normalize the gene expression levels relative to the internal controls.

**Table 2:** The sequence of primers.

	Sequence
TRIM38	Forward: 5′-CTCAAGAGCCACATCCTGGAAC-3′
	Reverse: 5′-GTTCCAAGGAGACAGCCTCTGA-3′
MITA	Forward: 5′- GGTGCCTGATAACCTGAGTATG-3′
	Reverse: 5′-GTTGCTGTAAACCCGATCCTTG-3′
GAPDH	Forward: 5′-GTCTCCTCTGACTTCAACAGCG-3′
	Reverse: 5′-ACCACCCTGTTGCTGTAGCCAA-3′

The primer sequences of TRIM38, MITA and GAPDH.

### Extraction of supernatants and cell components

The cell supernatants were collected in sterile EP tubes. Trichloroacetic acid (TCA, Sinopharm Chemical Reagent Co., Ltd.) was added at a ratio of 1:10 and mixed well. The mixture was placed in a 4 °C refrigerator overnight and then transferred to a 4 °C low-temperature centrifuge at 12,000 × *g* for 10 mins. The upper liquid was discarded, and the protein precipitate at the bottom of the tube was gently washed with 1 ml of sterilized PBS. The PBS was then removed, and 60–100 µl of western & IP lysate was added to dissolve the precipitate, depending on the amount of precipitation. Next, 5× loading buffer was added, and the sample was heated in a 95 °C water bath for 10 mins.

### Western blotting

Cell lysis was performed using western & IP lysates (Beyotime, Shanghai, China) for cells and RIPA lysate (GBCBIO Technologies, Guangzhou, China) for tissues. The protease inhibitor cocktail (Solarbio, Beijing, China) was added at a ratio of 1:100. Following lysis, the cells or tissues were transferred into tubes designed for cell or tissue crushing. Subsequently, they were centrifuged at 12,000 × *g* for 15 min at a temperature of 4 °C. The resulting supernatants were extracted separately and mixed with 5× loading buffer. For protein denaturation, the samples were boiled at 95 °C for 10 min. Afterwards, the collected samples were subjected to electrophoresis on a 10% SDS‒PAGE gel at a voltage of 80 V for 2 h. Following electrophoresis, the proteins were transferred to a membrane using a current of 350 mA for 2 h. For prevention of nonspecific binding, the membrane was blocked with 5% skim milk for 1 h and subsequently incubated overnight at 4 °C with the appropriate primary antibodies. The western blot bands derived from cells or tissues were visualized using an enhanced chemiluminescence (ECL, Millipore, USA) developer and a chemiluminescence imaging system (Shanghai Qinxiang Scientific Instrument Co., Ltd.).

The primary antibodies used in this study are as follows: rabbit monoclonal [EPR13130-55] antibody to MITA (#239074, Abcam, USA), mouse monoclonal antihuman TRIM38 (#MA5-26235, Invitrogen), rabbit monoclonal antibody [EPR19672] to caspase-1 (#207802, Abcam, USA), rabbit monoclonal antibody [EPR20829-408] to cleaved N-terminal GSDMD (#215203, Abcam, USA), rabbit monoclonal antibody [EPR19829] to GSDMD (#210070, Abcam, USA), and rabbit monoclonal antibody [EPR26492-84] to cGAS (#302617, Abcam, USA). Rabbit anti-GAPDH (#181602, Abcam, USA) was used as the internal control. IgG (HRP) goat anti-rabbit antibody (#6721, Abcam, USA) and IgG (HRP) goat anti-mouse antibody (#6789, Abcam, USA) were used as the secondary antibodies.

### Coimmunoprecipitation (Co-IP)

Endogenous Co-IP was performed on decidual tissues and M1 and M2 macrophages using western & IP lysates (Beyotime, Shanghai, China).

For immunoprecipitation, monoclonal antibodies against MITA (#239074, Abcam, USA) were utilized to immunoprecipitate tissue and cell lysates. For exogenous coimmunoprecipitation (Co-IP), 293 T cells were collected 24 h after transfection and subsequently lysed using a buffer containing 25 mM Tris-HCl (pH 7.4), 150 mM NaCl, 1% NP-40, 0.25% sodium deoxycholate, 1 mM EDTA, and a proteinase inhibitor cocktail (Solarbio, Beijing, China). Whole cell lysates were subjected to immunoprecipitation using monoclonal antibodies against Flag (#2368, CST, USA), HA (#236632, Abcam, USA), or His (#12698, CST, USA) in the presence of magnetic beads (MCE, USA). For both endogenous and exogenous Co-IP, normal rabbit IgG (#2729, CST, USA) was employed as a negative control. For prevention of nonspecific interference, all lysates were incubated with 10 µl of magnetic beads (MCE, USA) on a rotating shaker at 4 °C for 2 h. Generally, 1–2 μg of commercial antibody was added to 500 μl of tissue or cell lysates, and the mixture was incubated overnight at 4 °C. The immunocomplexes captured on the affinity gel or magnetic beads were thoroughly washed with lysis buffer and subsequently eluted with SDS loading buffer by boiling for 5 min. Subsequently, the samples were subjected to SDS‒PAGE and analyzed by western blotting.

### Immunohistochemical staining of TRIM38 and MITA in decidual tissues

The collected decidual tissues were fixed in 4% paraformaldehyde and subsequently embedded in paraffin. A microtome (HM355; Microm) was used to cut sections (4 μm), which were then deparaffinized through a series of incubations: 10 min in xylene, followed by 100%, 96%, and 70% ethanol, and deionized water. Antigen retrieval was performed using citrate buffer (pH 6.0), and the sections were incubated with 3% hydrogen peroxide for 30 min to remove endogenous peroxidase. For staining, the sections were blocked with 3% bovine serum albumin at room temperature for 30 min and then incubated overnight at 4 °C with the following primary antihuman antibodies: rabbit monoclonal antibody [EPR13130-55] to MITA (#239074, Abcam, dilution 1:4000) and mouse monoclonal antihuman TRIM38 (#MA5-26235, Invitrogen, dilution 1:150). After three washes with phosphate-buffered saline for 5 min each, the sections were incubated with the corresponding secondary antibodies (goat anti-rabbit antibody, 1:400, #5220-0336, SeraCare; goat anti-mouse antibody, 1:400, #5220-0341, SeraCare) for 1 h at room temperature. For visualization of the expression of MITA and TRIM38 in the decidua, diaminobenzidine chromogen was utilized. Finally, the sections were counterstained with haematoxylin.

### Triple immunofluorescence staining of decidual tissues

The obtained decidual tissues were fixed using 4% paraformaldehyde and subsequently embedded in paraffin. With a microtome (HM355; Microm), sections with a thickness of 4 μm were obtained. These sections were then deparaffinized by treatment with xylene for 10 min, followed by sequential washing with 100%, 96%, and 70% ethanol, as well as deionized water. Antigen retrieval was performed using citrate buffer (pH 6.0), and endogenous peroxidase was removed by incubating the sections with 3% hydrogen peroxide for 30 min. For triple staining of CD86 + CD206 and TRIM38, the sections were treated with 3% bovine serum albumin at room temperature for 30 min, followed by overnight incubation at 4 °C with the primary antihuman antibody CD206 (#60143-1-Ig, Proteintech, dilution 1:400). After the sections were washed three times with PBST, each for 5 min, a secondary antibody (1:400, #5220-0341, SeraCare) was applied and incubated at room temperature for 1 h. Subsequently, the sections were washed with PBST three times for 5 min each and incubated with Alexa Fluor™ 488 tyramide for 30 min at room temperature. Then, the sections were treated again for antigen retrieval and endogenous peroxidase quenching, followed by blocking. After that, CD80 staining was performed by incubating the sections with the corresponding primary antibody against CD80 (#bs-1035R, 1:200, Bioss), secondary antibody (#5220-0341, 1:400, SeraCare) and Cy3 tyramide. Similarly, staining of TRIM38 was detected by incubation with the corresponding primary antibody against TRIM38 (#334BAA80, 1:100, Invitrogen), secondary antibody (#5220-0341, 1:400, SeraCare), and Cy5 tyramide. The nucleus was stained with DAPI.

A similar protocol was used for the triple staining of CD86 + CD206 and MITA, where after staining of CD86 and CD206, the staining of MITA was detected by incubation with the corresponding primary antibody against MITA (#ab239073, 1:100, Abcam), secondary antibody (#5220-0336, 1:400, SeraCare), and Cy5 tyramide. The nucleus was stained with DAPI. Signal analysis was carried out using fluorescence microscopy (Olympus BX50) and digitally photographed.

### Statistical analysis

All statistical analyses were performed using GraphPad Prism 9 (GraphPad Software, La Jolla, USA). All the data are presented as the mean ± standard deviation. Significant differences between/among different groups were assessed using unpaired t test or one-way ANOVA followed by Bonferroni’s multiple comparison tests. *P* < 0.05 was considered statistically significant.

### Supplementary information

Supplementary figure - Revised

supplementary figure legends

Full and uncropped western blots

aj-checklist

## Data Availability

All the data are available upon reasonable request from the corresponding authors.
