# Supplementary figures and images for: Investigation into the role of the MITA-TRIM38 interaction in regulating pyroptosis and maintaining immune tolerance at the maternal-fetal interface
Source: Cell Death Dis. 2023 Nov 28;14(11):780. doi: 10.1038/s41419-023-06314-w (PMC10682411; doi:10.1038/s41419-023-06314-w)

Supplement Figure S1.

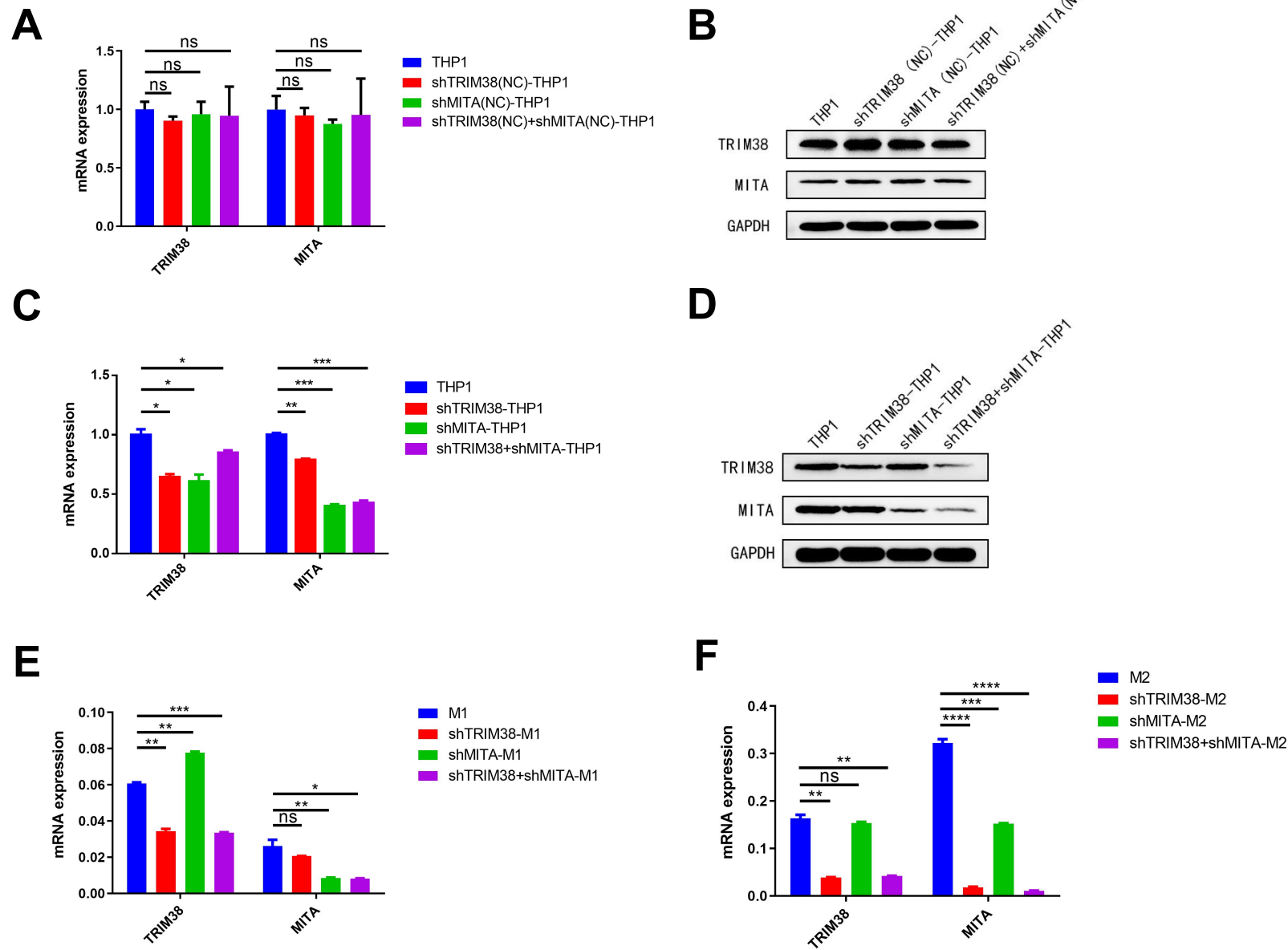

Supplement: Supplementary file 1 — Supplementary figure - Revised [file 41419_2023_6314_MOESM1_ESM.pdf]
