# Supplementary material for: Investigation into the role of the MITA-TRIM38 interaction in regulating pyroptosis and maintaining immune tolerance at the maternal-fetal interface
Source: Cell Death Dis. 2023 Nov 28;14(11):780. doi: 10.1038/s41419-023-06314-w (PMC10682411; doi:10.1038/s41419-023-06314-w)
Supplement: Supplementary file 2 — supplementary figure legends [file 41419_2023_6314_MOESM2_ESM.docx]

**Supplemental Figure S1. Differential expression of TRIM38 and MITA in the THP1 cell line constructed using control shRNA lentiviral particles or shRNA lentiviral particles at the mRNA and protein levels.** **(A)** Differences in the mRNA expression levels of TRIM38 and MITA in the THP1 cell line constructed using control shRNA lentiviral particles: shTRIM38(NC)-THP1 cells constructed using TRIM38(NC) lentiviral knockdown alone, shMITA (NC)-THP1 cells constructed using MITA lentiviral knockdown alone, and shTRIM38(NC)+shMITA(NC)-THP1 cells constructed with simultaneous TRIM38 and MITA lentiviral knockdown. **(B)** Differences in the protein expression levels of TRIM38 and MITA in the indicated cell lines: THP1, shTRIM38(NC)-THP1, shMITA(NC)-THP1, and shTRIM38(NC)+shMITA(NC)-THP1. **(C)** Differences in the mRNA levels of TRIM38 and MITA in the THP1 cell line and shTRIM38-THP1 cells constructed using TRIM38 lentiviral knockdown alone, shMITA-THP1 cells constructed using MITA lentiviral knockdown alone, and shTRIM38+shMITA-THP1 cells constructed with simultaneous TRIM38 and MITA lentiviral knockdown. **(D)** Differences in the protein expression levels of TRIM38 and MITA in the indicated THP1, shTRIM38-THP1, shMITA-THP1, and shTRIM38+shMITA-THP1 cell lines. **(E)** Differences in the mRNA expression levels of TRIM38 and MITA after and before M1 macrophages using lentiviral knockdown constructs. **(F)** Differences in the mRNA expression levels of TRIM38 and MITA after and before M2 macrophages using lentiviral knockdown constructs. **P* < 0.05, ***P* < 0.01, ****P* < 0.001, ns = nonsignificant.
