# Supplementary material for: Investigation into the role of the MITA-TRIM38 interaction in regulating pyroptosis and maintaining immune tolerance at the maternal-fetal interface
Source: Cell Death Dis. 2023 Nov 28;14(11):780. doi: 10.1038/s41419-023-06314-w (PMC10682411; doi:10.1038/s41419-023-06314-w)

**Figure 1.**

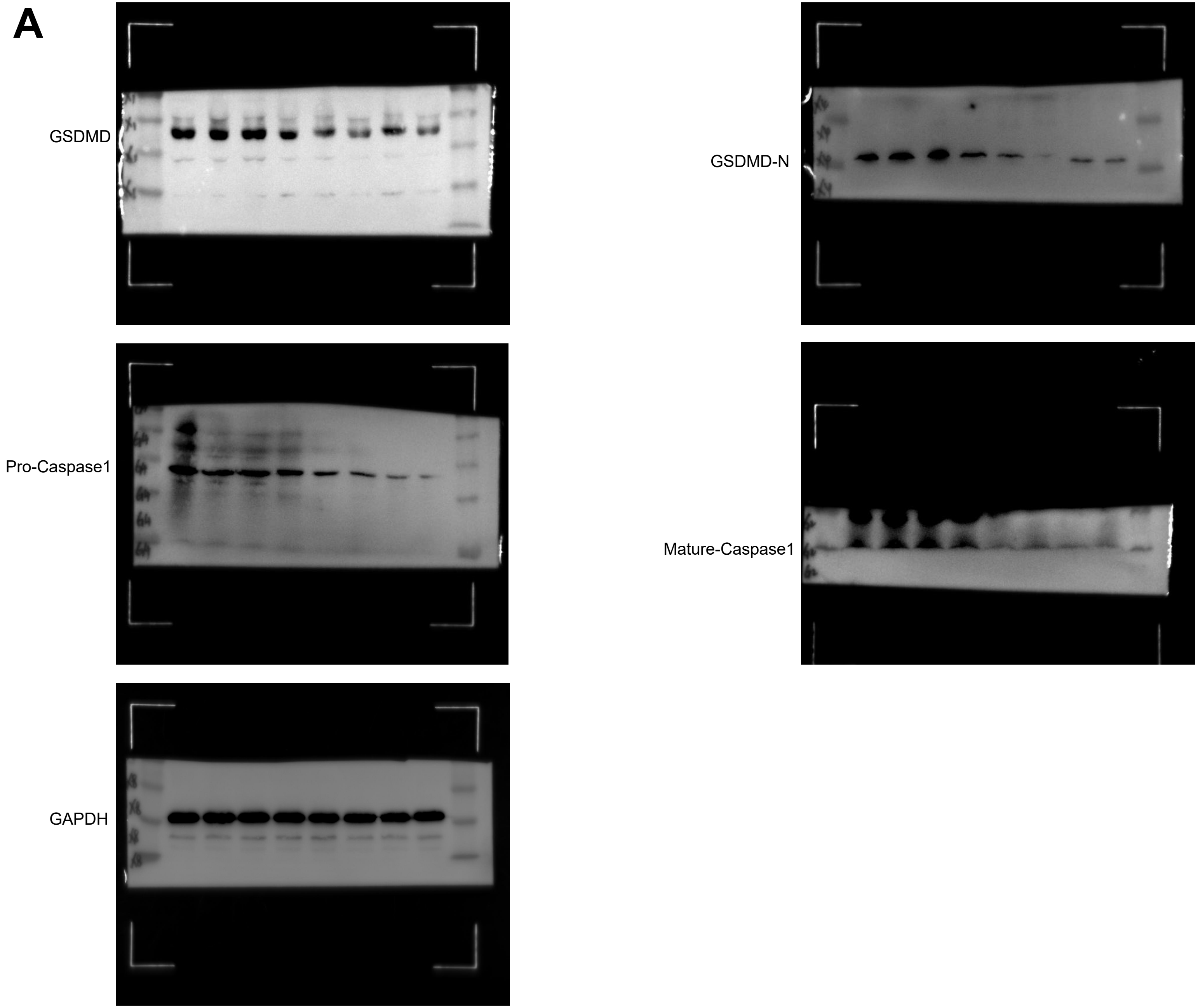

**Figure 3.**

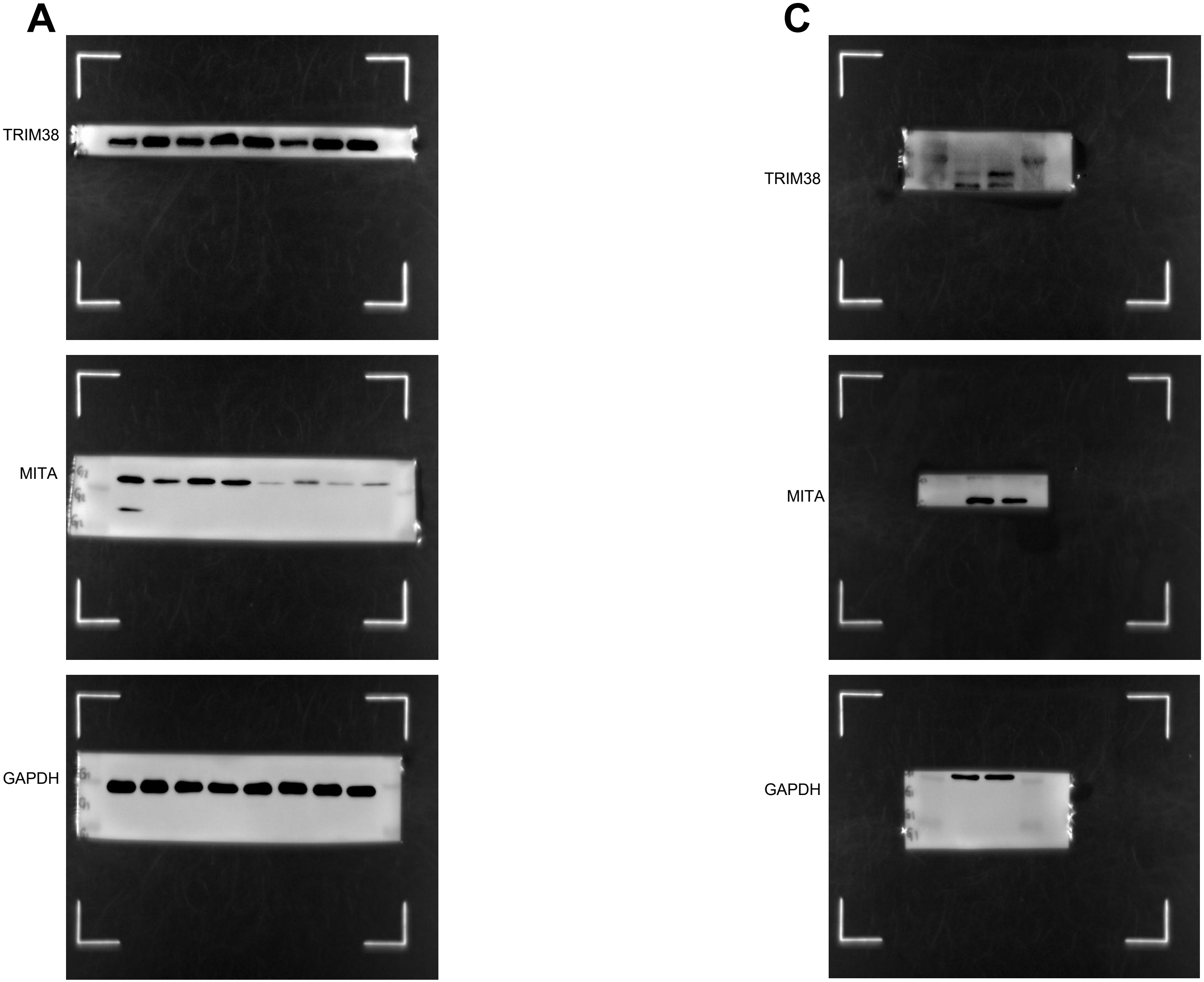

**Figure 4.**

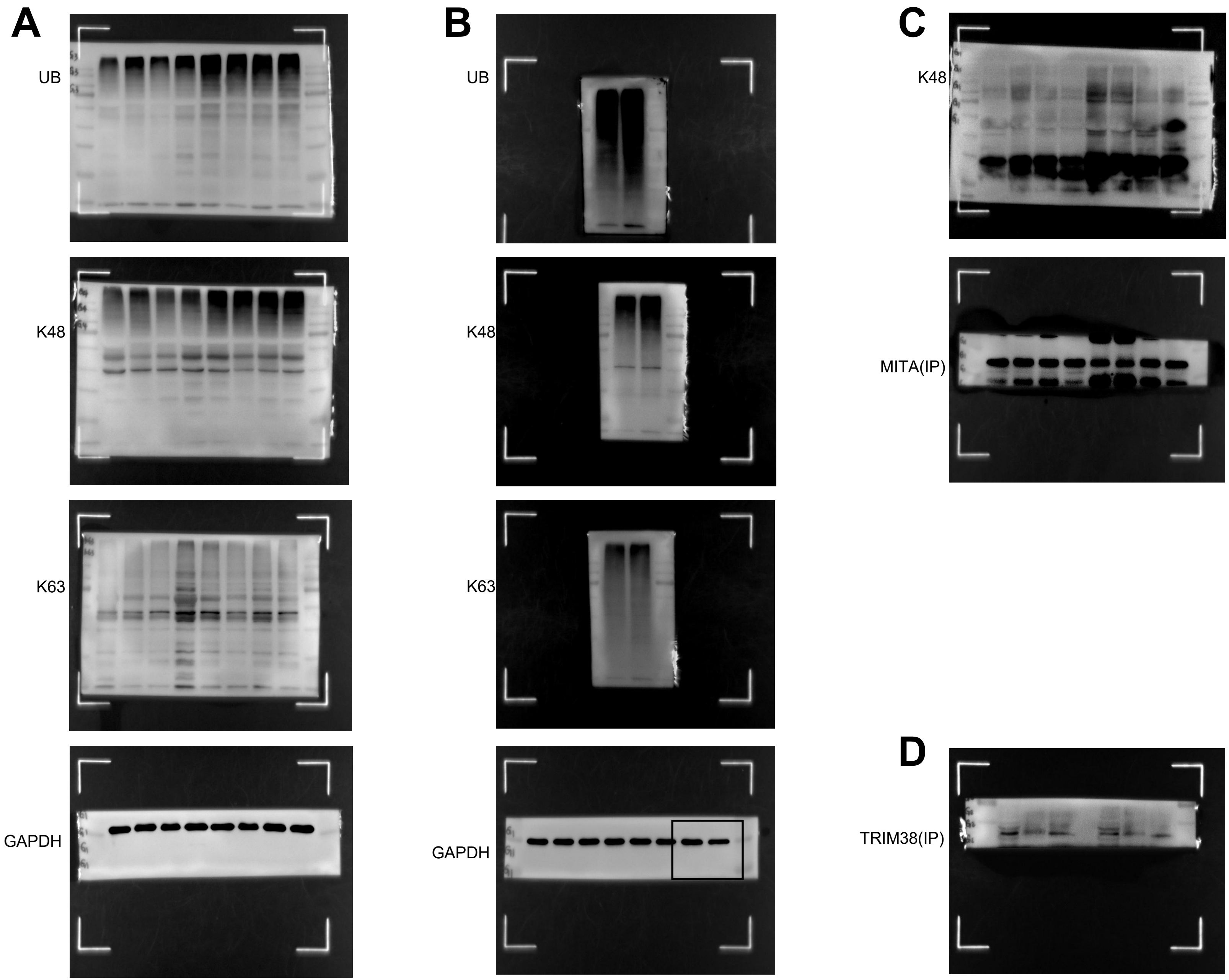

**Figure 5.**

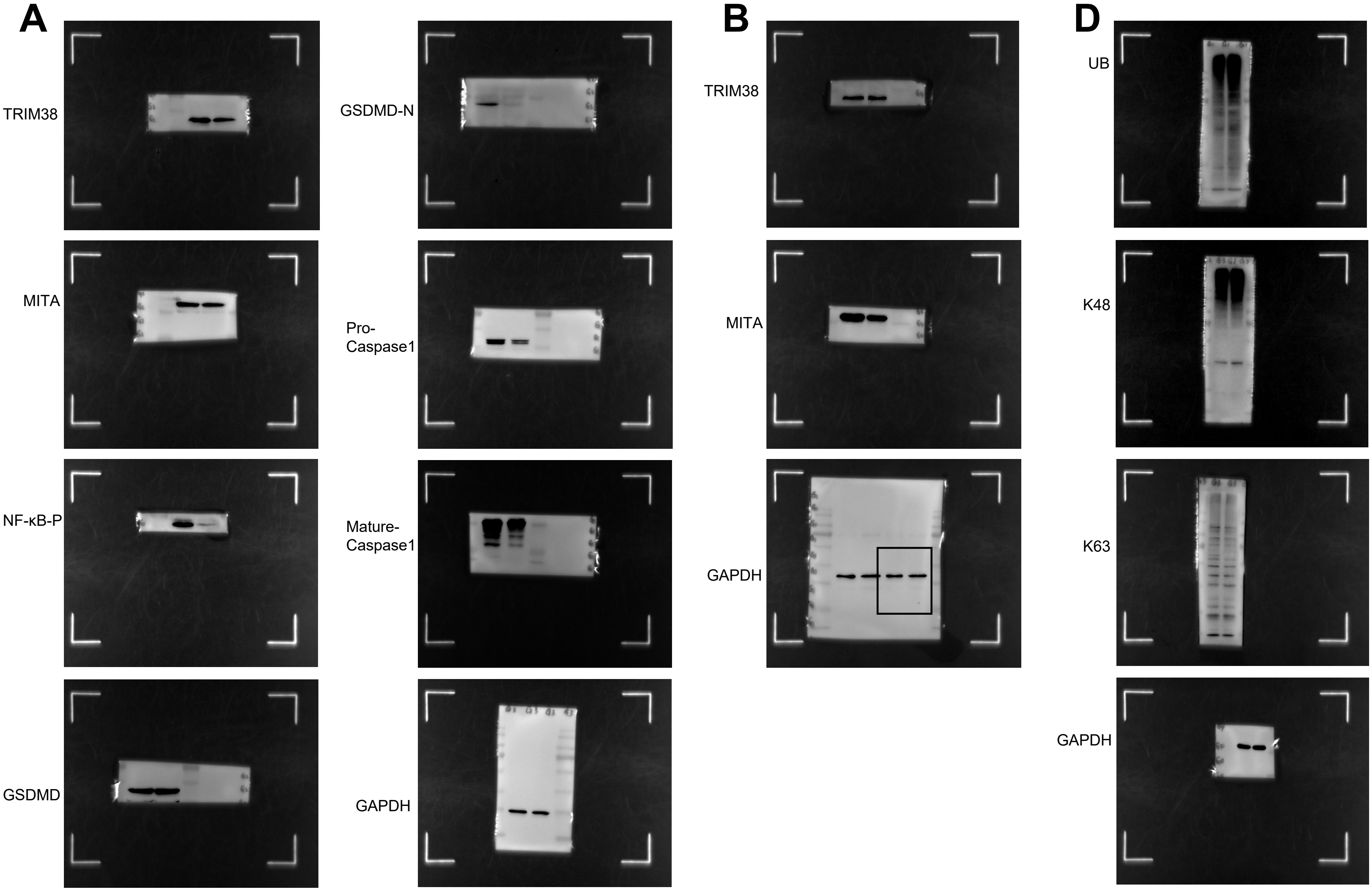
A
TRIM38
MITA
NF-κB-P
GSDMD
GSDMD-N
Pro-Caspase1
Mature-Caspase1
GAPDH
B
TRIM38
MITA
GAPDH
D
UB
K48
K63
GAPDH

**Figure 5.**

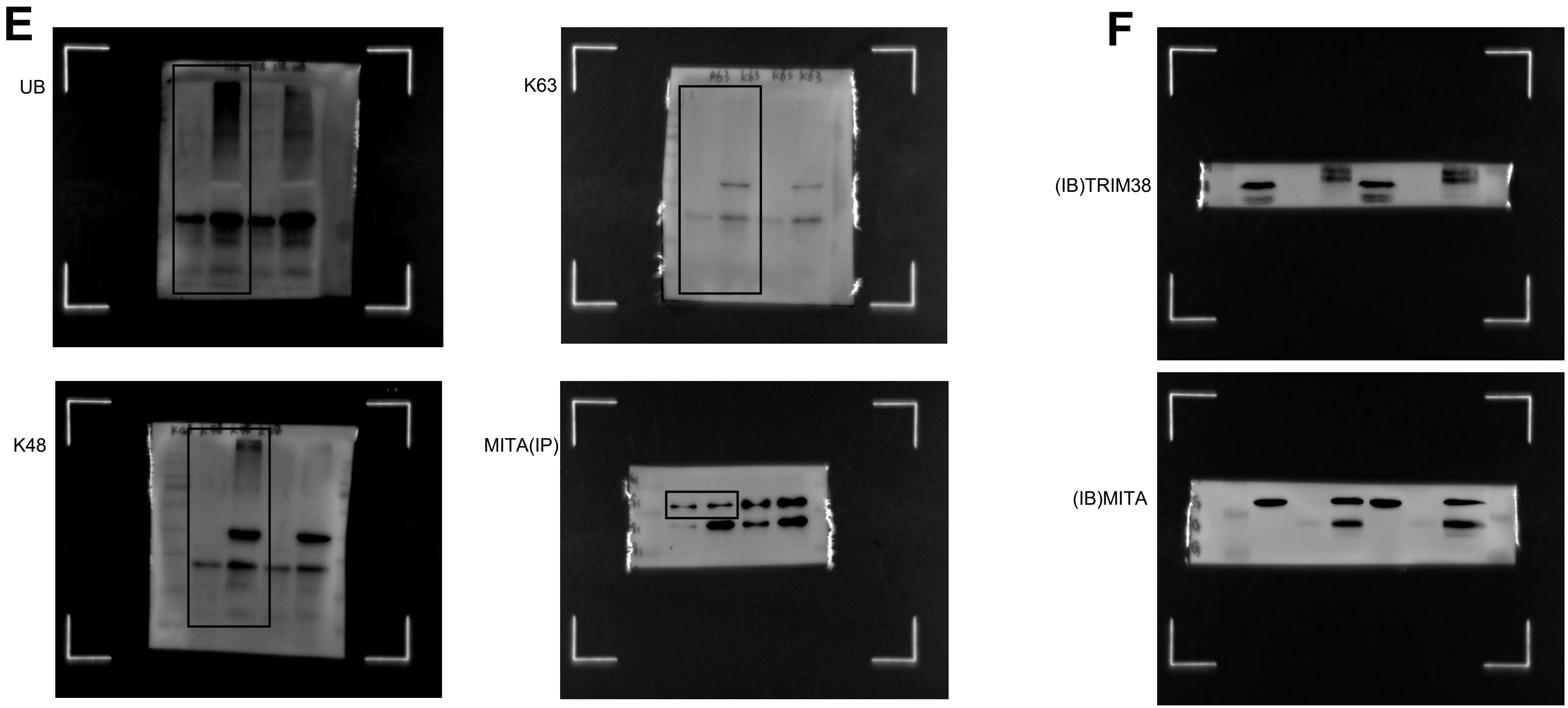

**Figure 6.**

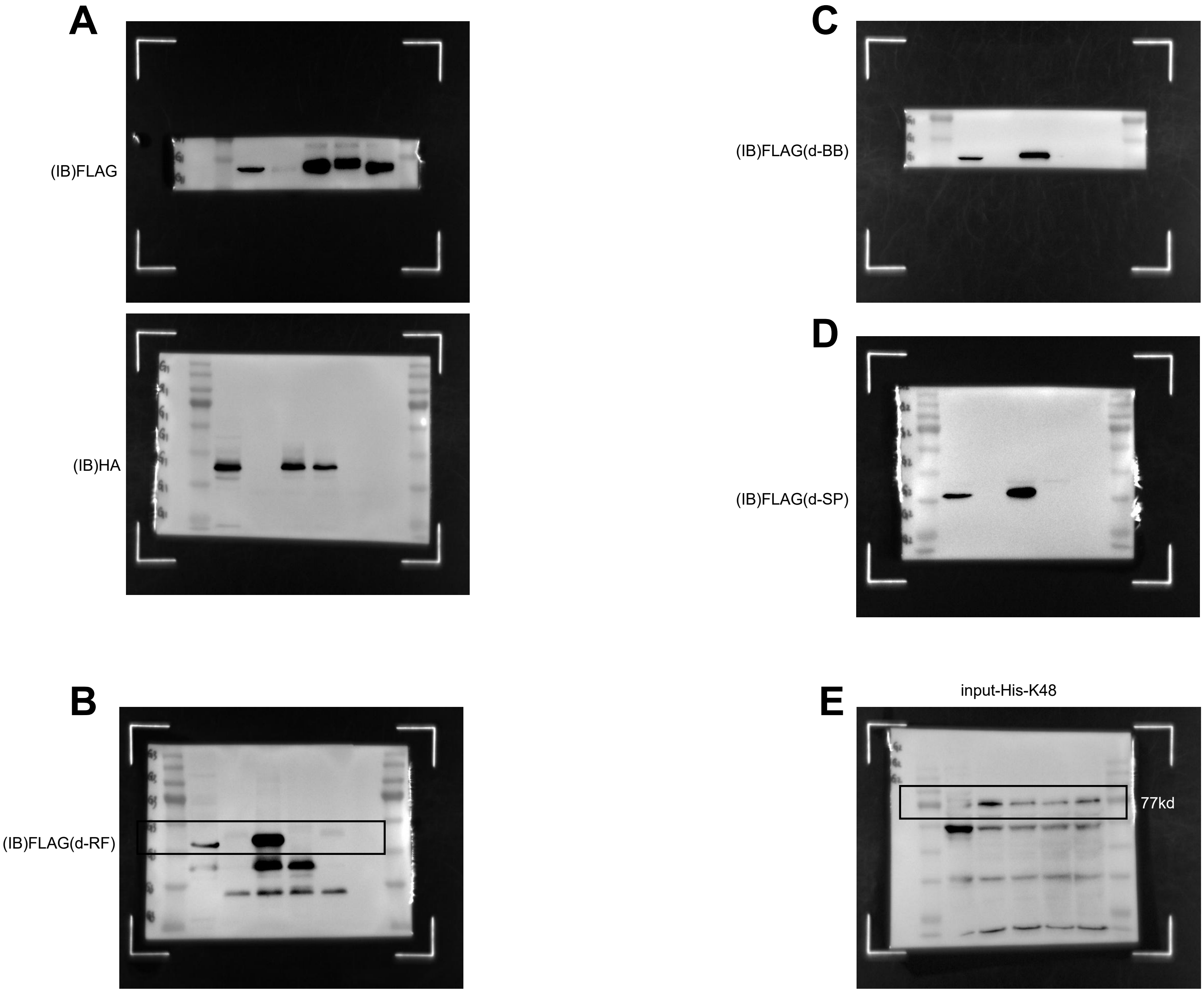
A
(IB)FLAG
(IB)HA
B
(IB)FLAG(d-RF)
C
(IB)FLAG(d-BB)
D
(IB)FLAG(d-SP)
E
input-His-K48
77kd

**Figure 7.**

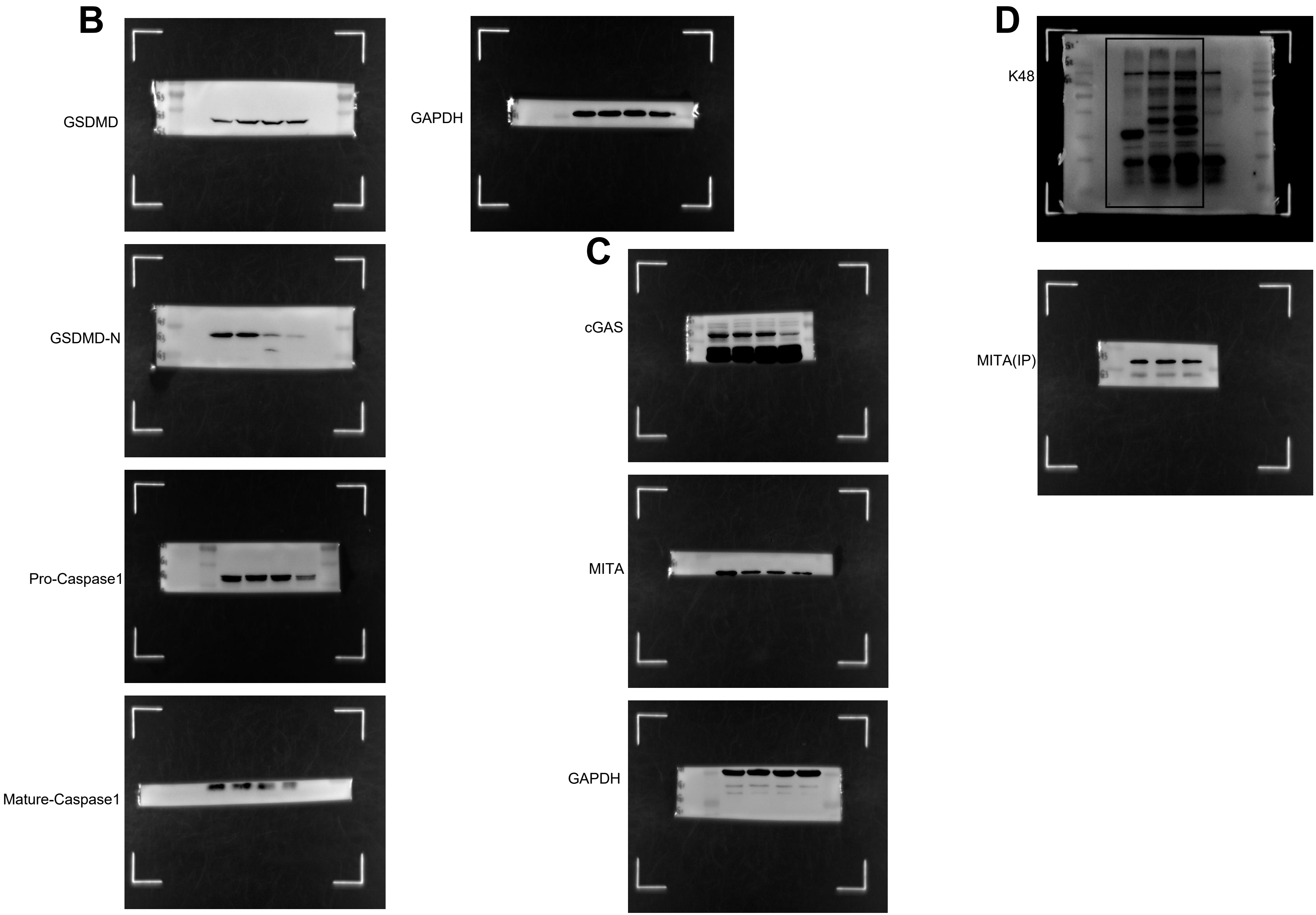
B
GSDMD
GAPDH
GSDMD-N
Pro-Caspase1
Mature-Caspase1
C
cGAS
MITA
GAPDH
D
K48
MITA(IP)

Figure 8.

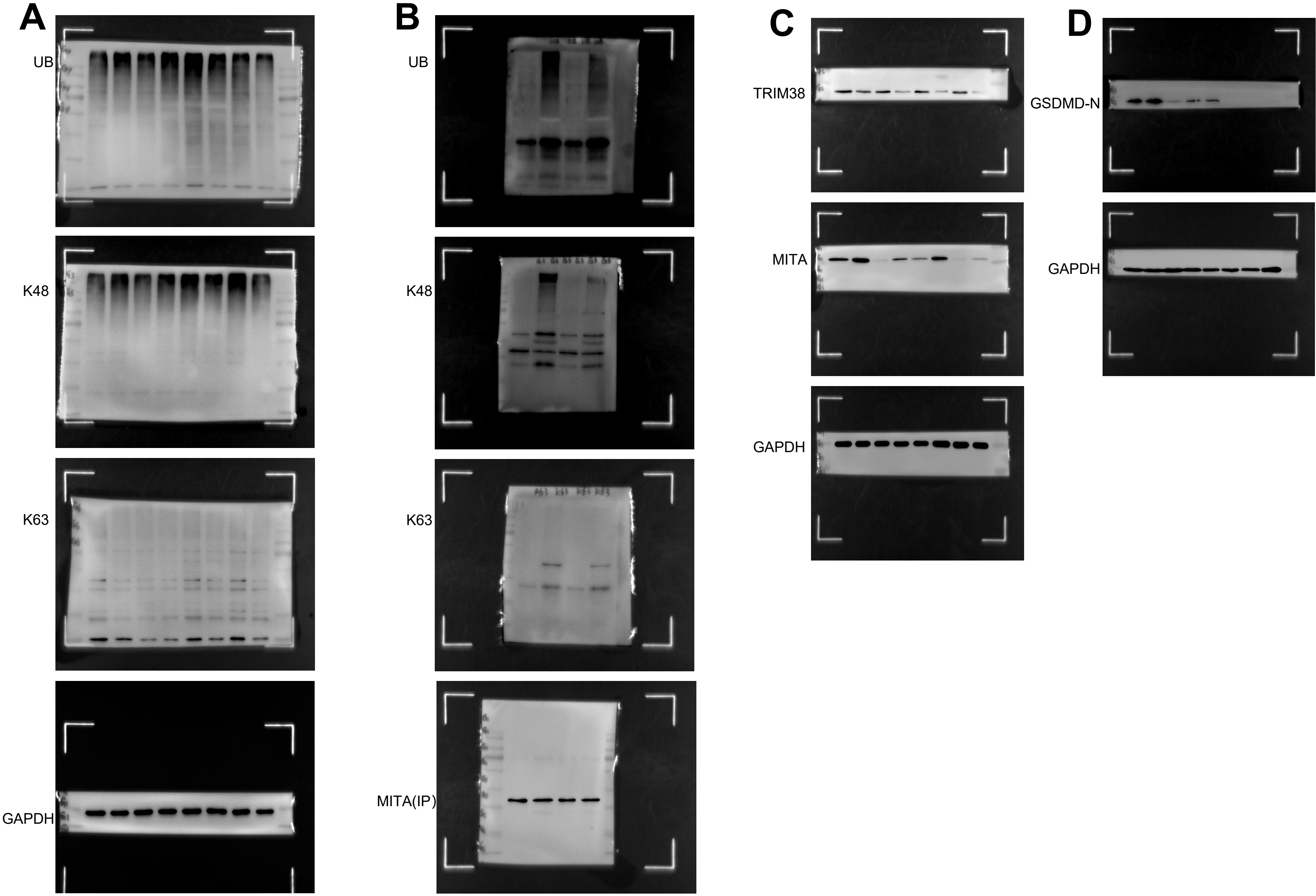
A
UB
K48
K63
GAPDH
B
UB
K48
K63
MITA(IP)
C
TRIM38
MITA
GAPDH
D
GSDMD-N
GAPDH

**Supplement Figure S1.**

B

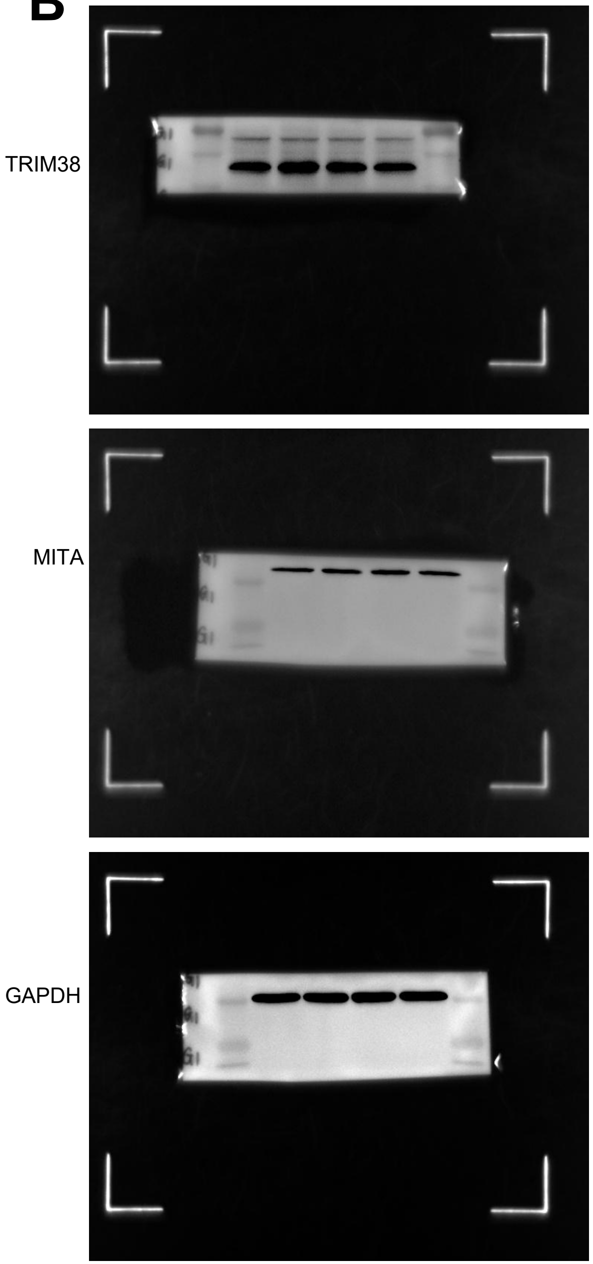


D

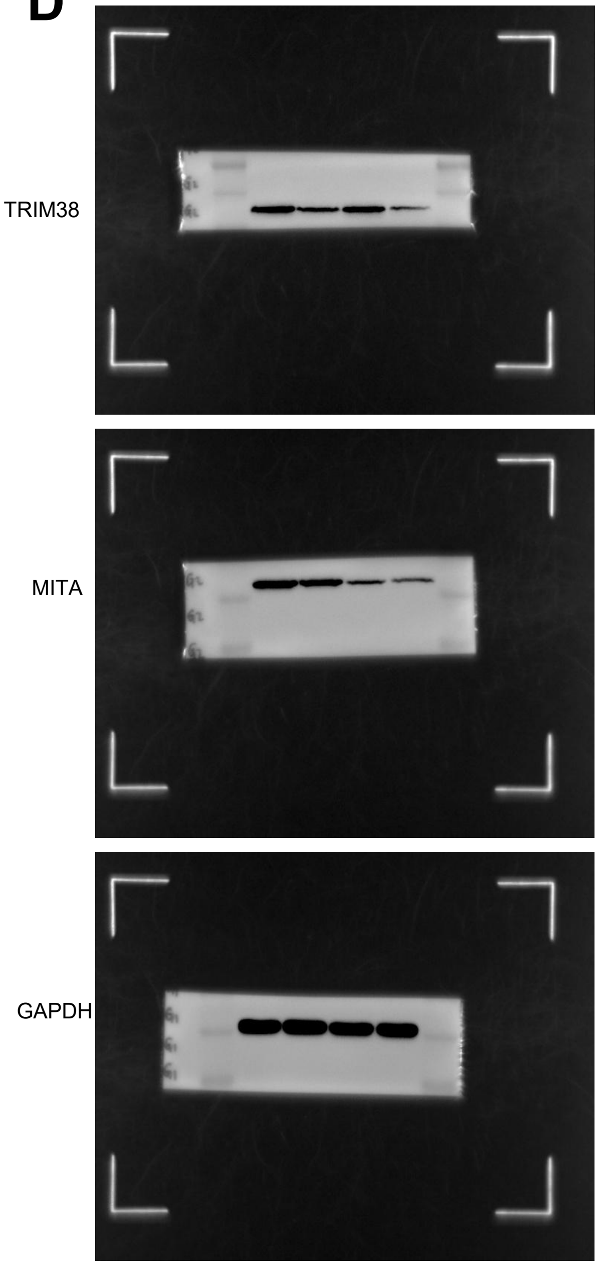

Supplement: Supplementary file 3 — Full and uncropped western blots [file 41419_2023_6314_MOESM3_ESM.pdf]
